# Comprehensive characterization of the *PeMADS* gene family in *Phyllostachys edulis* reveals new insights into floral development and evolution

**DOI:** 10.3389/fpls.2026.1806675

**Published:** 2026-04-21

**Authors:** Yue Zhu, Siyao Wang, Donglv Shu, Wentao Huang, Zeyi Ouyang, Shuoli Zheng, Changan Hu, Shoujin Cao, Song Sheng

**Affiliations:** 1 College of Forestry, Central South University of Forestry and Technology, Changsha, China; 2 Flower Research Institute, Hunan Botanical Garden, Changsha, China

**Keywords:** floral development, gene expansion, *MADS-box*, *Phyllostachys edulis*, RNA-seq analysis

## Abstract

*MADS-box* genes are pivotal regulators of plant growth, development, and stress responses. While previous studies have identified only 42 *MADS-box* genes (PeMADS) in *Phyllostachys edulis* (moso bamboo), the full scope of the *MADS* gene family in bamboo remained largely unexplored. In this study, we used phylogenetic analysis, motif and CDD analysis, integration of 16 RNA-seq datasets, and qPCR of moso bamboo-specific genes to characterize PeMADS genes, identifying a total of 110 *PeMADS* genes in moso bamboo. These genes were classified into two main types based on their structural features: type 1 genes, which contain only the *MADS* domain (PF01486), and type 2 genes, which harbor both the *MADS* and K-box domains (PF01486), suggesting a more complex role in gene regulation. Leveraging a large-scale integration of 16 RNA-seq datasets representing diverse developmental stages and stress treatments, we identified a distinct group of 15 type 2 *PeMADS* genes that are exclusively expressed in flower florets, highlighting their potential involvement in floral development. Additionally, through the analysis of these RNA-seq data, we uncovered a set of candidate genes that are likely regulated by *PeMADS*, providing further insights into their downstream regulatory networks. Finally, an evolutionary comparison of the *PeMADS* gene family across four different bamboo species revealed both conserved and divergent patterns, offering valuable insights into the evolution of *MADS-box* genes in the bamboo lineage. This study significantly enhances our understanding of the *PeMADS* gene family and lays the groundwork for future functional genomics studies and breeding applications in bamboo.

## 1 Introduction

Bamboo species exhibit diverse and often unpredictable flowering cycles, occurring at extended intervals and presenting significant challenges to ecosystems and agricultural systems. These cycles, characterized by mass flowering events followed by plant mortality, exert profound effects on forest dynamics, nutrient cycling, and biodiversity. Research has demonstrated that the gregarious flowering of bamboo species, such as *Chusquea culeou* in Patagonia, disrupts biogeochemical cycles by slowing litter decomposition and reducing nitrogen turnover, thereby altering carbon and nutrient dynamics (Austin and Marchesini, 2012). Similarly, studies on *Schizostachyum dullooa* in northeastern India have revealed that flowering and subsequent die-back facilitate canopy tree regeneration by creating ecological gaps but hinder bamboo population recovery due to high seedling mortality and limited colonization capacity (Das et al., 2019). These findings underscore the complex ecological ramifications of bamboo flowering, highlighting its dual role in regenerating ecosystems and destabilizing existing vegetation patterns. The periodicity of bamboo flowering is believed to be regulated by genetic and environmental factors, with hypotheses such as the “lunar clock” proposing that bamboo species retain cellular memory of sun–moon phasing to determine flowering intervals. Evidence for this has been noted in species like *Phyllostachys pubescens* and *Fargesia qinlingensis*, where flowering cycles are synchronized to specific lunar conditions (Clerget, 2021). Mast flowering, a strategy in which bamboo species synchronize flowering events across vast regions, has been hypothesized as an evolutionary mechanism to overwhelm seed predators and enhance pollination success, adding complexity to ecosystem dynamics (Keeley and Bond, 1999). These extended flowering intervals, spanning decades to more than a century, have been associated with slower molecular evolution rates in temperate woody bamboos, suggesting a link between reproductive periodicity and evolutionary constraints (Ma et al., 2017).

Central to the molecular mechanisms of bamboo flowering are the *MADS-box* genes, a conserved family of transcription factors that play essential roles in floral organ identity, reproductive development, and flowering time regulation. Their involvement in bamboo mirrors findings in other plant species, emphasizing their evolutionary significance and widespread conservation. In rice, for instance, 75 *MADS-box* genes have been associated with reproductive development and stress responses, while in the tea plant, 136 members of this gene family have been characterized for their roles in floral organogenesis and abiotic stress response (Arora et al., 2007; Zhang et al., 2018b) Genome-wide analyses in *Phyllostachys edulis* and *Dendrocalamus latiflorus* have identified *MADS-box* genes linked to critical hormonal regulation pathways, including auxin and gibberellin, essential for floral organ development and pollen viability (Yang et al., 2023). These findings resonate with the ABC model of floral organ identity described in *Arabidopsis thaliana*, which demonstrates how combinations of *MADS-box* genes dictate floral organ formation (Coen and Meyerowitz, 1991). Phylogenomic analysis across plant genomes has revealed the diversification of *MADS-box* genes into distinct functional clades, facilitated by gene duplication events, enabling these genes to acquire specific developmental roles such as floral patterning, seed development, and environmental adaptation (Becker and Theissen, 2003; Gramzow and TheiSsen, 2013).

The evolutionary conservation of these genes in monocots like bamboo, rice, and *Brachypodium* underscores their central role in shaping reproductive success across perennial grasses, while the identification of bamboo-specific regulatory elements highlights unique adaptations to sporadic and extended flowering cycles. Such findings establish bamboo as an informative model for studying perennial grass flowering mechanisms, bridging evolutionary biology with practical applications in crop improvement and perennial grass management. This intricate balance of conservation and diversification reflects the duality of *MADS-box* genes, which are fundamental in understanding the molecular drivers of bamboo flowering and exploring broader agricultural applications. Building on this understanding, the study of bamboo flowering is critical for decoding the biological mechanisms underlying its unique reproductive cycle, characterized by prolonged vegetative phases, synchronized mass flowering events, and subsequent plant mortality.

Transcriptomic analyses of bamboo species, including *P. edulis* and *D. latiflorus*, have revealed key genes involved in floral transitions, with *MADS-box* genes such as *PeMADS5* emerging as critical regulators. The overexpression of *PeMADS5* in model systems such as *A. thaliana* has demonstrated its ability to induce early flowering and alter floral morphology, underscoring its pivotal role in bamboo reproductive development (Zhang et al., 2018b). Further genome-wide studies in *Dendrocalamus latiflorus* have identified differential gene expression patterns in developing flower buds, elucidating molecular pathways central to flowering and floral organogenesis (Zhang et al., 2012). Comparative genomic analyses have reinforced the conservation of key flowering pathways, involving genes such as FT and LFY, across bamboo and other monocots while also identifying bamboo-specific regulatory elements linked to its sporadic and prolonged flowering intervals (Biswas et al., 2016). The integration of environmental and genetic cues into flowering control mechanisms has been further demonstrated through stress-responsive genes and microRNAs, highlighting an adaptive interplay between abiotic signals and genetic regulation (Jiao et al., 2019)—for example, research on *Bambusa oldhamii* has revealed mutations in meiotic genes affecting pollen viability, alongside the conserved expression of flowering regulators, emphasizing the complexity of bamboo’s reproductive biology (Zhao et al., 2022). Hormonal regulation, particularly the balance between auxin and cytokinins, has also been shown to critically influence floral transitions as demonstrated in controlled induction studies using *in vitro* tissue culture.

Insights into reproductive regulation have been further advanced through degradome analyses of stamen-specific microRNAs, such as miR159, which regulate anther development and pollen fertility, providing a more nuanced understanding of bamboo’s reproductive mechanisms (Cheng et al., 2020). Similarly, studies on *Phyllostachys violascens* have revealed thousands of differentially expressed genes (DEGs) involved in stress responses and hormonal regulation during early flower induction, with *MADS-box* genes playing critical roles in organ development (Jiao et al., 2019). These findings align with transcriptomic analyses in *P. edulis*, which have elucidated intricate gene networks and microRNAs central to floral transitions, offering a comprehensive understanding of the molecular frameworks governing bamboo flowering (Gao et al., 2014). Efforts to circumvent bamboo’s prolonged vegetative phases have utilized *in vitro* techniques, with plant growth regulators such as cytokinins effectively inducing early flowering, thereby facilitating reproductive studies and hybridization strategies (John and Nadgauda, 1999).

Comparative analyses across monocots, including rice and *Brachypodium*, have highlighted conserved pathways regulating flowering time, such as photoperiod and vernalization mechanisms, while identifying bamboo-specific adaptations to its extended flowering cycles (Biswas et al., 2016). Environmental triggers, such as drought and stress, have also been implicated in flowering regulation, with transcriptomic evidence linking these factors to hormonal and circadian rhythm pathways (Jiao et al., 2019). Additionally, microRNAs such as miR159, which regulate stamen differentiation and fertility, underscore the complex genetic and molecular basis of bamboo reproduction (Cheng et al., 2020). Despite significant progress, the genetic mechanisms governing bamboo’s irregular flowering cycles remain incompletely understood, as historical studies have documented substantial variability across species, reflective of complex evolutionary adaptations (Zheng et al., 2020). Addressing these challenges through integrative approaches that combine omics technologies with functional validation of candidate genes is imperative to unravel bamboo’s flowering mechanisms. Such research not only advances the understanding of bamboo’s reproductive biology but also offers a robust framework for genetic engineering aimed at improving flowering regulation, crop resilience, and sustainable cultivation practices.

The importance of studying *MADS-box* genes in bamboo stems from their pivotal roles in regulating flowering time, floral organ development, and the transition from vegetative to reproductive phases. These processes are crucial to bamboo’s ecological and economic significance. Bamboo’s unique reproductive patterns, which range from sporadic flowering to synchronous gregarious events often followed by mass die-offs, necessitate an in-depth investigation into the molecular mechanisms driving these phenomena. Comprehensive genome-wide studies in species such as *P. edulis* have identified 42 *MADS-box* genes, which are integral to floral organ differentiation and the vegetative-to-reproductive transition. Functional studies have demonstrated the ability of key genes, such as *PeMADS5*, to induce early flowering and alter floral morphology, underscoring the functional conservation of *MADS-box* genes across monocots, including rice, and their critical roles in reproductive success (Zhang et al., 2018b). Investigations into MIKC^c^-type *MADS-box* genes in *Dendrocalamus latiflorus* have further elucidated their association with hormonal regulation pathways, including auxin, gibberellin, and jasmonic acid, which are essential for floral organogenesis and pollen viability (Yang et al., 2023). Comparative transcriptomic studies have confirmed the conservation of these genes in the ABCDE model of floral organogenesis, as observed in *Oryza sativa* and *A. thaliana*, validating bamboo’s relevance as a model for monocot floral research (Shih et al., 2014). Moreover, the functional characterization of *AGAMOUS-like* genes, such as *DlMADS18* in *D. latiflorus*, has revealed their influence on flowering time and morphological traits, offering insights into evolutionary adaptations and practical applications in crop breeding (Bo et al., 2005). These findings align with broader research in grass species, which emphasizes the integration of *MADS-box* gene expression into developmental and environmental regulatory networks, such as photoperiod and vernalization responses (Wysocki et al., 2016).

The study of bamboo *MADS-box* genes not only addresses the biological challenges posed by its irregular flowering cycles but also provides a robust framework for genetic engineering aimed at improving flowering regulation, crop resilience, and sustainable cultivation practices. By bridging evolutionary insights with functional genomics, this research contributes to a broader understanding of plant developmental pathways and offers novel solutions to ecological and agricultural challenges. These advances enhance the understanding of bamboo reproductive biology while informing strategies for genetic improvement, sustainable cultivation, and ecosystem management, ensuring the viability of this economically and ecologically significant plant group. Through these interconnected findings, the shared conservation and unique adaptations of *MADS-box* genes in bamboo emerge as a focal point, providing critical insights into the molecular and evolutionary basis of flowering in perennial grasses.

MADS-box transcription factors constitute one of the most important regulatory gene families controlling plant development, particularly floral organ specification and reproductive development. The classical ABC model of flower development has provided a fundamental framework for understanding how combinations of MADS-box genes determine floral organ identity (Bowman and Moyroud, 2024). Comparative genomic studies have revealed that MADS-box genes originated early in the evolution of green plants and subsequently diversified through gene duplication and functional specialization, giving rise to the large and complex gene family observed in modern plant genomes (Han et al., 2025; Zhang et al., 2024). These transcription factors play crucial roles not only in floral organ formation but also in various developmental processes and stress responses.

Bamboo species exhibit a unique and still poorly understood flowering behavior characterized by extremely long flowering intervals and synchronous flowering events across large geographic regions. This unusual reproductive strategy often leads to large-scale die-off of bamboo populations, resulting in significant ecological and economic consequences (Wu et al., 2024). Despite the ecological importance of bamboo flowering, the molecular mechanisms underlying floral transition and development in bamboo remain largely unclear. Therefore, genome-wide characterization and evolutionary analysis of key flowering regulatory genes, such as the MADS-box gene family, are essential for improving our understanding of bamboo reproductive biology.

## 2 Materials and methods

### 2.1 Multi-step homolog search of *MADS* genes in bamboo

The genome sequences of *P. edulis* (moso bamboo) and *A. thaliana* were obtained from GigaDB (http://gigadb.org/dataset/view/id/100498/File_page/2) and Ensembl Plants (https://plants.ensembl.org/index.html), respectively. The genome sequences of *Bonia amplexicaulis*, *Guadua angustifolia*, *Olyra latifolia*, and *Raddia guianensis* were retrieved from the Bamboo Genome Database (http://gigadb.org/dataset/view/id/100498/File_page/2) (Guo et al., 2019). To identify *MADS-box* genes, the Hidden Markov Model (HMM) profiles of the *MADS* domain (PF00319) and K-box domain (PF01486) were obtained from the Pfam protein family database (Finn et al., 2011; Jaina et al., 2020). The HMMER Search function in TBtools-II (v2.443) was used to identify genes containing only the *MADS* domain and those containing both the *MADS* and K-box domains. An E-value threshold of E <0.01 was applied, and duplicate sequences were removed after manual curation. The same procedure was applied to identify *MADS-box* genes in *B. amplexicaulis*, *G. angustifolia*, *O. latifolia*, and *R. guianensis*.

### 2.2 Phylogenetic analysis and conserved motif prediction

Phylogenetic relationships among the identified *MADS-box* genes in *P. edulis* and *A. thaliana* were constructed using MEGA11 (V11.0.13) software. Multiple sequence alignments were performed using the MUSCLE algorithm, and phylogenetic trees were generated using the NJ (neighbor-joining) method with the following parameters: 5,000 bootstrap replicates, p-distance method, partial deletion for gaps, and a site coverage cutoff of 50%. The resulting trees were visualized and annotated using the iTOL tool (iTOL: Interactive Tree Of Life) (Letunic and Bork, 2007). Conserved motifs within the *MADS-box* genes were identified using the MEME suite (Bailey et al., 2009), with the maximum number of motifs set to 10.

### 2.3 Classification and physicochemical characterization of *MADS-box* genes

The *MADS-box* genes of *P. edulis* were systematically classified into subfamilies based on the phylogenetic relationships. Sequential identifiers (*PeMADS1* to *PeMADS110*) were assigned according to their evolutionary clustering. Physicochemical properties, including molecular weight and *pI* (isoelectric point), were analyzed using the Protein Parameter Calc function in TBtools.

### 2.4 Transcriptome analysis of *MADS-box* genes under developmental and stress conditions

Raw RNA sequencing data were retrieved from the NCBI Sequence Read Archive (SRA, https://www.ncbi.nlm.nih.gov/sra, accessed on January 1, 2024) using the SRA Toolkit-prefetch (version 2.8.0). A total of 16 RNA-seq datasets for *P. edulis* were analyzed to explore the expression profiles of *MADS-box* genes under various developmental stages and stress conditions, including bamboo seedlings that were treated with cold stress (SRP193861) (Liu et al., 2020), bamboo leaves that were treated with drought and salt stress (SRP311022) (Xie et al., 2019), bamboo roots that were treated with nitrogen addition (SRP355516) (Zhu et al., 2023), bamboo roots that were treated with cadmium stress (SRP379766) (Chen et al., 2016), bamboo roots that were treated with NAA hormone (SRP109631) (Wang et al., 2017), bamboo leaves that were treated with salicylic acid and abscisic acid (SRP311022_1) (Xie et al., 2019), bamboo’s different tissue-specific expression profiles (SRP094812) (Zhao et al., 2018), bamboo’s various growth stages (SRP165714) (Zhang et al., 2021a), bamboo shoots of different varieties (SRP067720), different regions of bamboo shoots (SRP087658) (Gamuyao et al., 2017), bamboo roots that were treated with GA_3_ (SRP119416) (Zhang et al., 2018a), 1.2-m-long shoot of dwarf bamboo (SRP133731) (Qiu et al., 2024), bamboo shoots of different heights (SRP200837) (Wang et al., 2019), lateral buds of bamboo rhizome (SRP237549) (Shou et al., 2020), bamboo shoots in spring and winter (SRP294609) (Wang et al., 2022), and bamboo 18th internode (SRP366087) (Zhang et al., 2021a).

The raw sequencing data in FASTQ format were first subjected to quality assessment using the FASTQC program (https://www.bioinformatics.babraham.ac.uk/projects/fastqc/) to evaluate Q20, Q30, GC content, and sequence duplication levels. High-quality reads were subsequently aligned to the *P. edulis* genome, downloaded from GigaDB (http://gigadb.org/dataset/view/id/100498/File_page/2), using Hisat2 (version 2.2.1) (Kim et al., 2015). The aligned reads were converted into gene and transcript expression values using fragment per kilobase of transcript per million mapped reads (FPKM).

### 2.5 Identification of *P. edulis*-specific *MADS-box* genes

To identify *P. edulis*-specific *MADS-box* genes, full-length MADS protein sequences from *B. amplexicaulis*, *G. angustifolia*, *O. latifolia*, and *R. guianensis* were merged into a single dataset as subject sequences. The *P. edulis MADS-box* genes were used as query sequences, and pairwise comparisons were performed using the Blast function in TBtools. Genes with an identity score ≤25% were initially selected, while those with identity >85% were excluded. Genes with identity values ≤85% were retained for further analysis. A Venn diagram was generated using TBtools to identify the overlap between genes meeting the ≤25% and ≤ 85% thresholds, which represented *P. edulis*-specific *MADS-box* genes. A heatmap was constructed to visualize the maximum identity values of these genes in comparison with *MADS-box* genes from the other bamboo species.

### 2.6 Quantitative real-time PCR analysis of differential genes in *P. edulis*

Quantitative real-time PCR (RT-qPCR) technology was employed to analyze the tissue-specific expression patterns of differential genes in *P. edulis*. Three-month-old robust seedlings with consistent growth vigor were selected, and samples were collected from three tissues, namely, roots, culms, and leaves. Each treatment was performed with three biological replicates. The samples were wrapped in tinfoil, labeled, immediately frozen in liquid nitrogen, and subsequently stored in a refrigerator at -80 °C.

Quantitative primers were designed using Primer 5 software (**Supplementary Table 1**), and their specificity was verified using the primer design tool of the National Center for Biotechnology Information (NCBI). The RT-qPCR reaction system (total volume) was as follows: 4 μL of diluted cDNA template, 5 μL of 2× ChamQ Universal SYBR qPCR Master Mix (Vazyme), and 0.5 μL each of forward and reverse primers. The RT-qPCR reaction program was set as follows: pre-denaturation at 95 °C for 3 min, followed by 38 cycles of denaturation at 95 °C for 10 s and annealing/extension at 60 °C for 30 s. Finally, a melting curve analysis was performed from 65 °C to 95 °C (holding at 65 °C for 5 s and at 95 °C for 15 s). Data were analyzed using the 2^-ΔΔct^ method, with the *NTB* gene of *P. edulis* serving as the reference gene.

### 2.7 Data analysis and network visualization

Various R packages (Langfelder and Horvath, 2008), including dplyr, ggplot2, reshape2, Rmisc, and stringr, were employed for data analysis, statistical processing, and graphical visualization. Network diagrams were constructed and visualized using Cytoscape (3.10.4) software to elucidate the relationships between co-expressed genes and potential regulatory interactions.

## 3 Results

### 3.1 Phylogenetic and functional characterization of the *MADS-box* gene family in moso bamboo

To gain insights into the genetic structure and potential functions of the MADS-box gene family in moso bamboo, a comprehensive bioinformatics analysis was conducted. This study combined homologous sequence alignment with HMM-based domain prediction to identify genes belonging to the MADS-box gene family within the moso bamboo genome. In total, 110 genes were identified, with 64 genes containing only the MADS domain (type_1) and 46 genes containing the K-box domain and the MADS domain (type_2).

**Figure 1:**
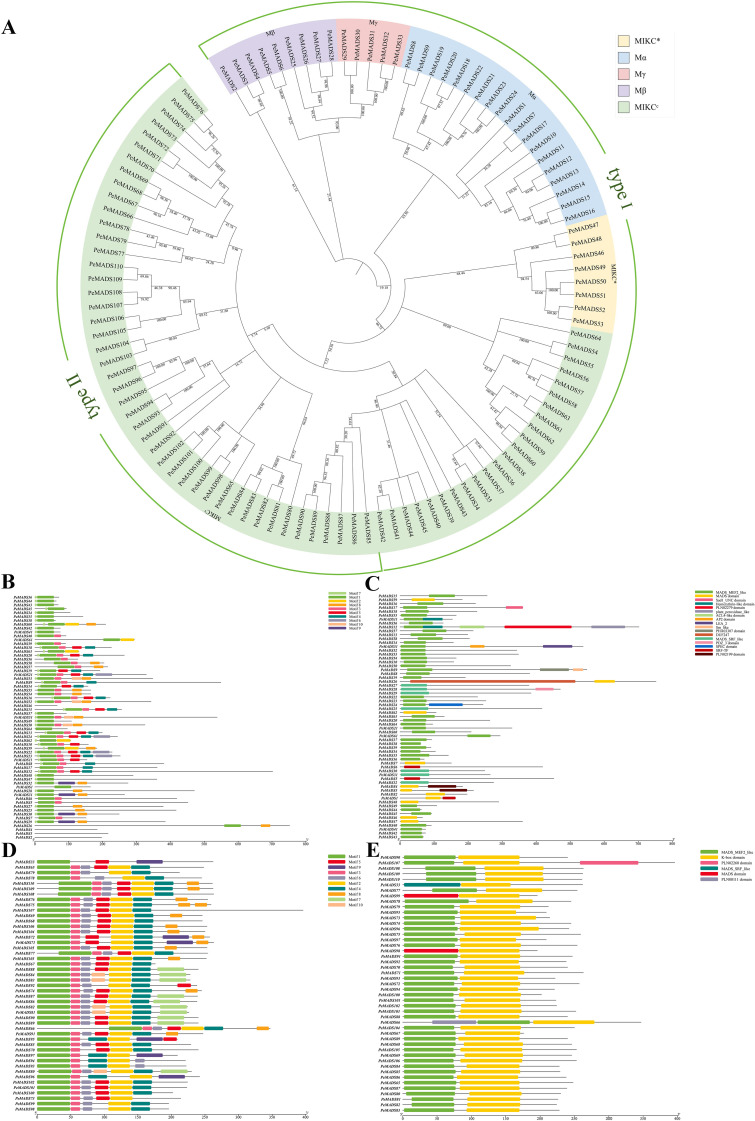
Phylogenetic analysis, conserved motif analysis, and domain visualization of MADS proteins in *Phyllostachys edulis*. Phylogenetic trees were constructed by using the neighbor-joining (NJ) method. **(B, C)** Conserved motif analysis of MADS proteins was performed using the MEME tool. **(A)** The phylogenetic tree can be divided into two parts, Type I and Type II, and color blocks of different colors represent different subfamilies. **(B)** Conserved motifs of type I MADS proteins in *P. edulis*, which only contain the MADS domain. **(C)** Conserved motifs of type II MADS proteins in *P. edulis* showing the conserved motifs of *P. edulis* proteins containing MADS and K-BOX domains. **(D)** Domain visualization of type I MADS proteins in *P. edulis*. **(E)** Domain visualization of type II MADS proteins in *P. edulis*.

A phylogenetic analysis of these genes was carried out using full-length MADS protein sequences from *O. sativa* and *A. thaliana* to further categorize them based on their domain structures (**Supplementary Figure 1**). The MADS domain genes in moso bamboo were classified into two major types: Type I, which only contains the MADS domain, and Type II, which contains both the K-box domain and the MADS domain. The Type I genes were further divided into four subfamilies (Mα, Mβ, Mγ, and MIKC*) and some genes of the MIKC^c^ subfamily; all Type II genes belong to the MIKC^c^ subfamily (**Figure 1A**). These findings revealed that the MADS domain was more diverse, with a greater number of subfamilies and a more complex phylogenetic structure. This complexity may imply that the MADS domain may perform more intricate and specialized regulatory roles compared to the K-box domain. Further investigation is required to fully clarify the functional divergence between these two domains.

To explore the functional roles of the MADS-box genes, the conserved motifs within these genes were analyzed using the MEME suite, based on the phylogenetic relationships. A total of 10 distinct conserved motifs were identified, with genes within the same subfamily exhibiting similar motif patterns, suggesting that these genes may share similar regulatory functions. The MADS gene family was categorized into two primary groups: one containing genes with only the MADS domain (PF01486, motif 1) (**Figure 1B**) and the other containing both the MADS and K-box domains (PF01486 and PF00319, motif 1 and motif 2) (**Figure 1D**). In the group with only the MADS domain, motifs 1, 3, 7, and 8 were commonly shared, while motif 10 was present in only a few genes. In the group with both domains, motifs 1–4 were frequently shared, while motifs 7 and 9 were found in only a subset of genes. Notably, some domains detected by the HMM model were not identified by MEME, which was likely due to the differences in the algorithmic approaches used by the two methods. Overall, the genes containing the K-box domain showed a higher degree of similarity in conserved motifs and exhibited a simpler structural organization, which may indicate that these genes are involved in regulating a broad range of cellular processes. In contrast, the genes containing the MADS domain displayed greater variability in conserved motifs and a more complex structure, suggesting that these genes may regulate more specific and intricate biological functions. These findings provide valuable insights into the functional diversification of the MADS-box gene family in moso bamboo, highlighting the distinct roles of the MADS and K-box domains in regulating diverse biological processes.

The conserved domains of MADS-box proteins in *P. edulis* were predicted using the NCBI CDD database and visualized by using TBtools. The results showed that Type I proteins (**Figure 1C**) mainly contained the N-terminal MADS-MEF2-like domain, and a few members possessed a small number of other additional domains, with a relatively simple structural composition. Type II proteins (**Figure 1E**) exhibited typical and conserved structural characteristics, including the N-terminal MADS-MEF2-like domain and the central K-box domain. In addition, the distribution positions of conserved domains were roughly consistent with those of conserved motifs, further indicating the high conservation of this protein family in sequence and structure.

Additionally, based on the subfamily classification and the size of the encoded proteins, the genes were renamed from *PeMADS1* to *PeMADS110*. A detailed analysis of their physicochemical properties, including *pI*, was also conducted (**Supplementary Table 2**).

### 3.2 Gene expression profiling of PeMADS genes in *P. edulis* under multiple environmental stresses

**Figure 2:**
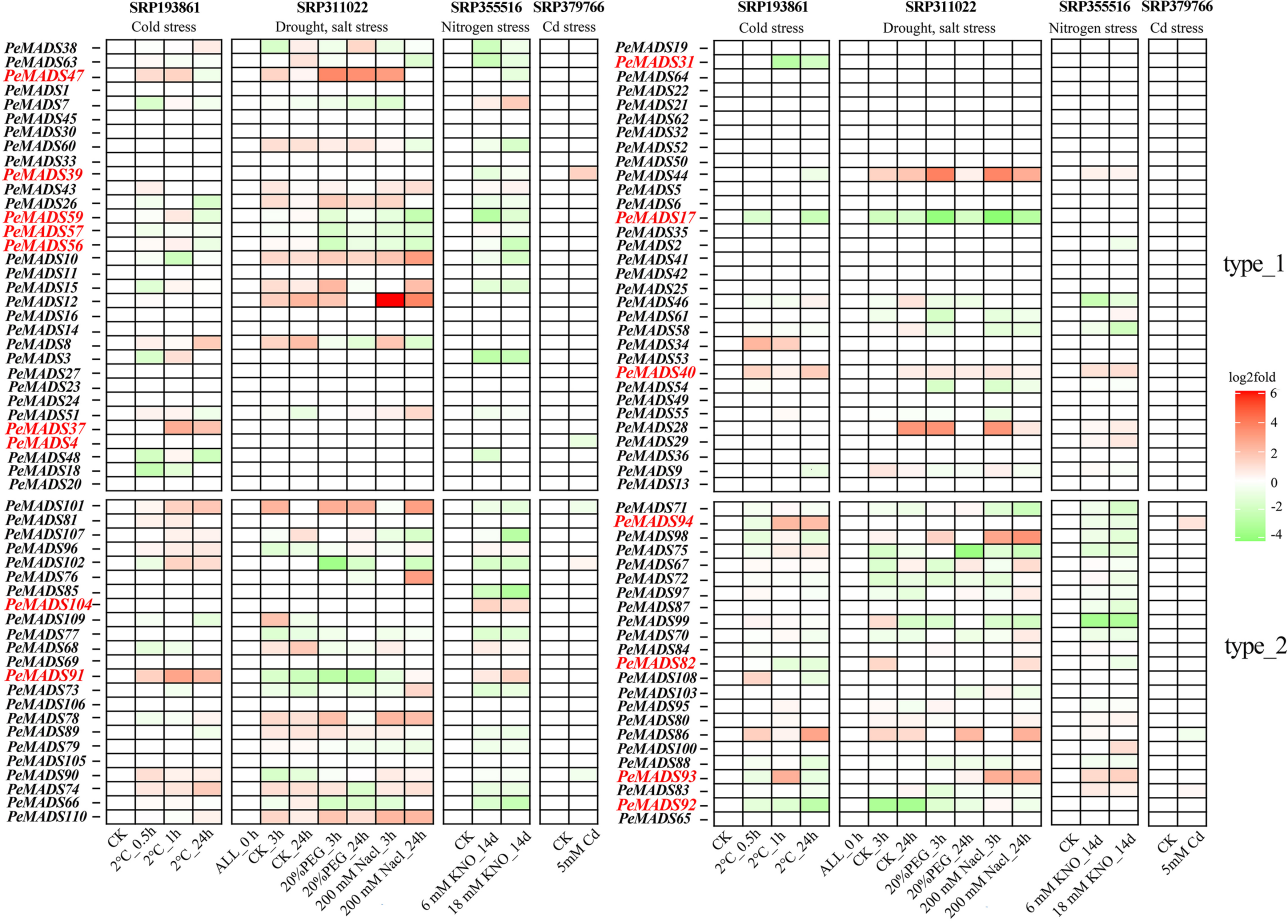
Gene expression heatmap of *MADS* gene to environmental stress in *Phyllostachys edulis*, with SRA numbers of different transcriptomes at the top, IDs of different genes on the ordinate, and different sample groups on the abscissa. The type_1 on the right represents genes containing only *MADS* domains, and the type_2 represents genes containing both *MADS* and *K-box* domains. Red represents higher gene expression levels, and green represents lower gene expression levels. The genes marked red on the ordinate are the significant genes discussed in the results. ALL_0 h means no processing will be done.

This study aims to investigate the gene expression responses of *P. edulis* to various environmental stressors, including cold, drought, salt, nitrogen, and heavy metal stresses, in order to identify key genes involved in stress resistance mechanisms. To achieve this, seedlings and roots of *P. edulis* were exposed to these stresses, and the expression of *PeMADS* genes was analyzed under different experimental conditions, such as varying durations of cold exposure, drought and salt stress treatments, nitrogen concentrations, and heavy metal exposure (**Figure 2**). The gene expression analysis revealed significant changes in several *PeMADS* genes, which appear to be crucial in regulating stress resistance. Under cold stress (-2 °C for 0, 0.5, 1, and 24 h), genes such as *PeMADS92*, *PeMADS82*, *PeMADS31*, and *PeMADS47* showed a decreased expression with prolonged cold exposure, while other *PeMADS* genes, including *PeMADS91*, *PeMADS94*, and *PeMADS37*, increased significantly. These findings indicate a differential response to cold stress. In drought and salt stress treatments, where drought was simulated with 20% PEG and salt with 200 mM NaCl, a similar trend was observed. The expression of *PeMADS17*, *PeMADS56*, *PeMADS57*, and *PeMADS59* decreased under both stresses, indicating that these genes may play key roles in stress resistance regulation. Under nitrogen stress (0, 6, and 18 M concentrations), genes *PeMADS93*, *PeMADS104*, and *PeMADS40* showed significant upregulation, highlighting their role in nitrogen adaptation. Finally, under heavy metal stress (5 M Cd for 60 days), *PeMADS94* and *PeMADS39* exhibited an increased expression, while *PeMADS4* decreased, further supporting the role of *PeMADS* genes in metal stress response. In summary, the K-box-domain-containing *PeMADS* genes showed significant changes in expression across various stress conditions, suggesting their central role in enhancing stress resistance in *P. edulis*. These findings provide valuable insights into the molecular mechanisms underlying stress adaptation in bamboo, which could inform future efforts to improve the stress resilience of this economically important species.

### 3.3 Expression dynamics of bamboo genes in response to hormonal treatments

**Figure 3:**
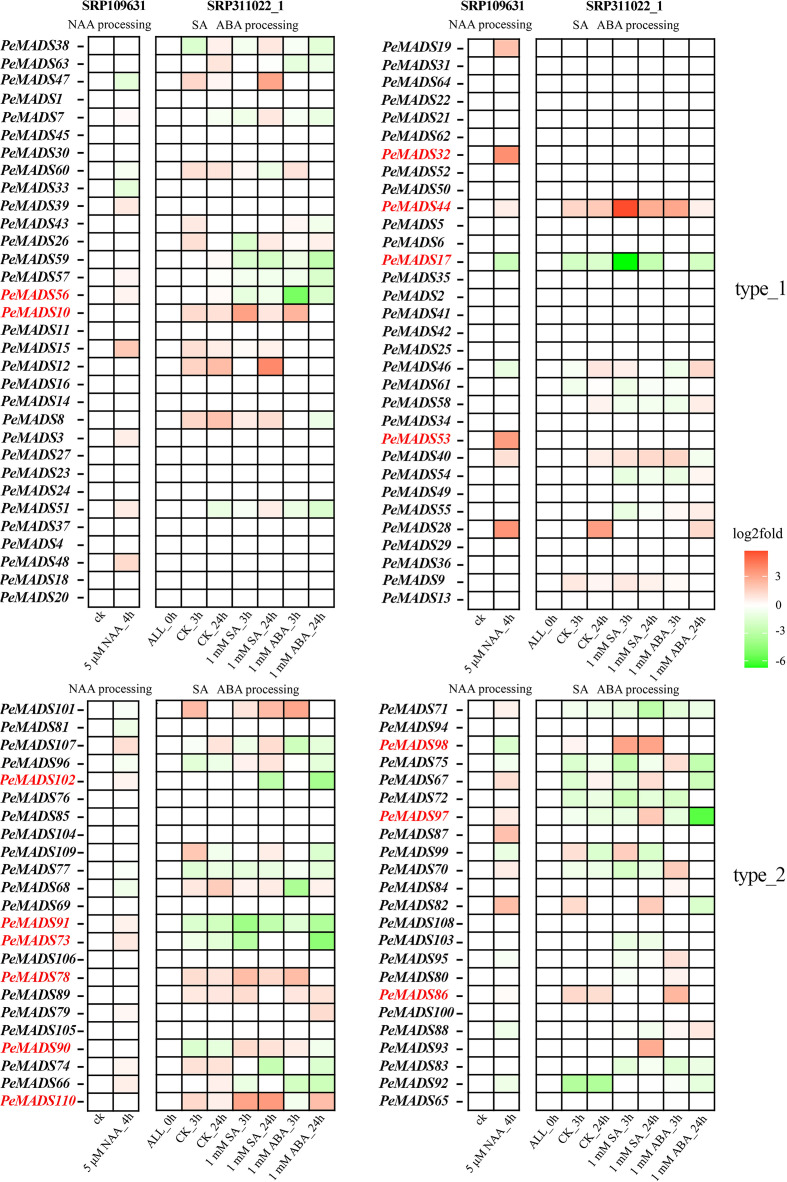
Gene expression heatmap of different plant hormones by *MADS* gene in *Phyllostachys edulis*. The SRA number of different transcriptomes is shown at the top, the IDs of different genes is on the ordinate, and the abscissa shows the different sample groups. The type_1 on the right represents genes containing only MADS domains, and the type_2 represents genes containing both MADS and K-box domains. Red represents higher gene expression levels, and green represents lower gene expression levels. The genes marked red on the ordinate are the significant genes discussed in the results. ALL_0 h means no processing will be done.

This study also intends to investigate the gene expression changes in bamboo seedlings under various plant hormone treatments to better understand the regulatory mechanisms involved in growth and stress responses (**Figure 3**). Bamboo seedlings were treated with 5 μM NAA (auxin) for 4 h, and root samples were collected. The results showed significant changes in the expression of genes *PeMADS98*, *PeMADS53*, *PeMADS32*, and *PeMADS17*, with *PeMADS98* and *PeMADS17* significantly downregulated, while the others were upregulated, indicating a complex regulatory network in response to auxin. In a separate experiment, bamboo leaves were treated with 1 M salicylic acid (SA) and 1 M abscisic acid (ABA) for 3 and 24 h, respectively. Under SA treatment, genes such as *PeMADS98*, *PeMADS110*, PeMADS90, *PeMADS44*, and *PeMADS10* showed an increased expression, while *PeMADS17*, *PeMADS102*, *PeMADS91*, and *PeMADS73* showed a decreased expression. Under ABA treatment, genes including *PeMADS86*, *PeMADS78*, *PeMADS44*, *PeMADS10*, *PeMADS56*, *PeMADS102*, *PeMADS91*, *PeMADS73*, and *PeMADS97* exhibited distinct responses, with *PeMADS86*, *PeMADS78*, *PeMADS44*, and *PeMADS10* upregulated, while others were downregulated. These results suggest that K-box-domain-containing genes show a more pronounced response to plant hormones like SA, ABA, and NAA, highlighting their critical role in regulating bamboo growth and stress responses and their involvement in broader regulatory networks governing bamboo’s physiological adaptations.3.4 Differential expression and functional dynamics of *MADS-box* genes in bamboo tissues and developmental stages.

**Figure 4:**
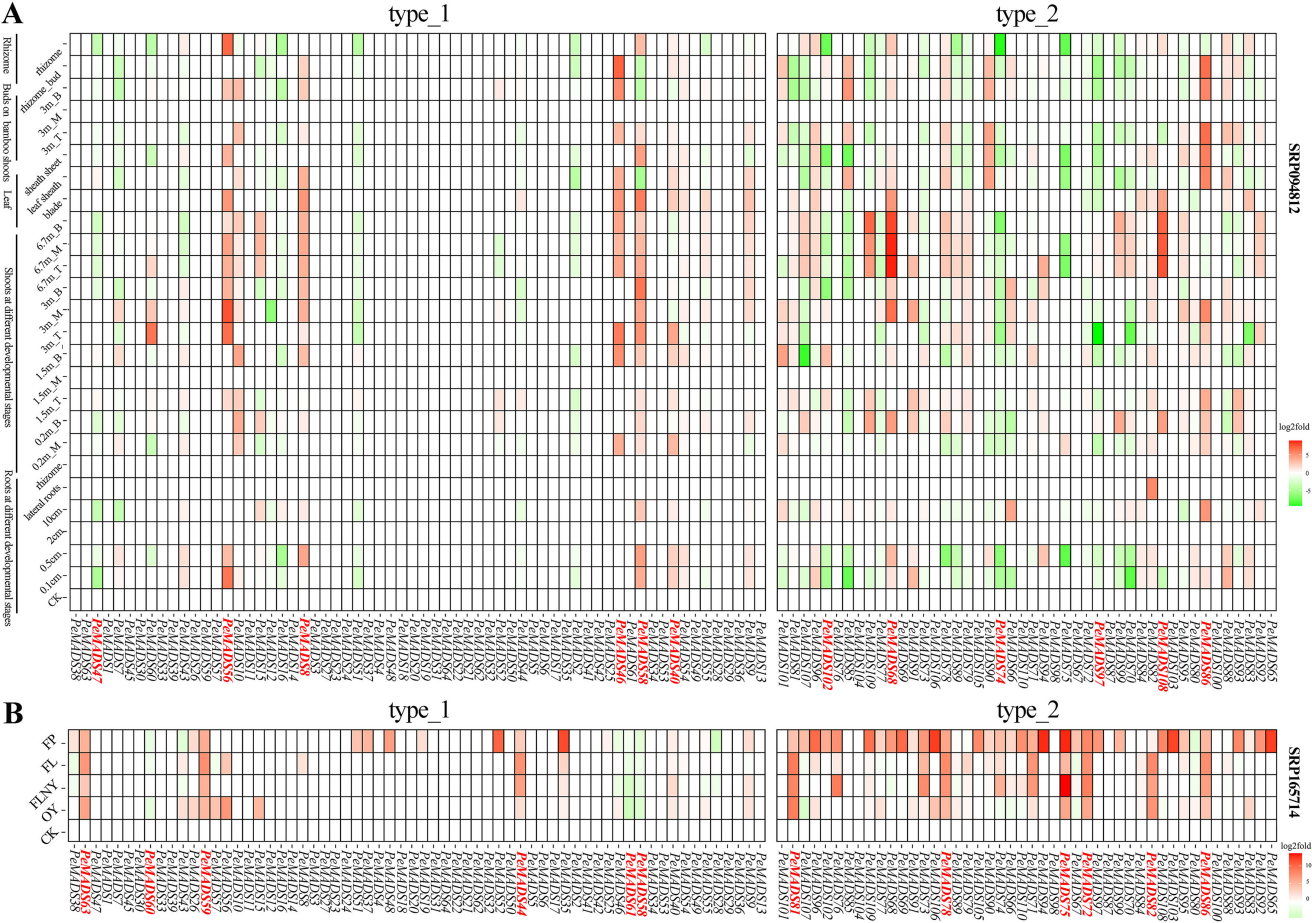
**(A)** Heatmap of *MADS* gene expression in different plant tissues of *Phyllostachys edulis*, with the SRA number of the transcriptome at the top, the IDs of different genes on the ordinate, and different sample groups on the abscissa. The type_1 on the right represents genes containing only MADS domains, and the type_2 represents genes containing both MADS and K-box domains. Red represents higher gene expression levels, and green represents lower gene expression levels. The genes marked red on the ordinate are the significant genes discussed in the results, where B represents the base, T represents the top, and M represents the middle. **(B)** *MADS* gene expression heatmap of different growth and development stages of *P. edulis*, the SRA number of the transcriptome at the top, the IDs of different genes on the ordinate, and different sample groups on the abscissa. The type_1 on the right represents the genes containing only the MADS domain, and the type_2 represents the genes containing both the MADS and K-box domains. Red represents the higher gene expression level, and green represents the lower gene expression level. OY, 1 year; FLNY, flower in the next year; FL, flower leaf; FP, flower florets.

To better understand the role of *MADS* genes in bamboo development, an analysis was conducted to examine their expression profiles across various plant tissues and during the transition from vegetative to reproductive growth (**Figure 4A**). The expression profiles of *MADS* genes across different plant tissues in bamboo were assessed, with the gene expression in Shoot_0.2m_T used as the baseline for comparison. Notably, several genes, including *PeMADS86*, *PeMADS108*, *PeMADS68*, *PeMADS40*, *PeMADS58*, *PeMADS46*, *PeMADS8*, and *PeMADS56*, exhibited higher expression levels across the majority of tissues. In contrast, genes such as *PeMADS97*, *PeMADS74*, *PeMADS85*, *PeMADS102*, and *PeMADS47* were found to have relatively lower expression levels in most tissues. It was observed that genes exhibiting high expression levels were predominantly associated with the *MADS* domain, while those containing the K-box domain generally showed lower expression levels, highlighting a differential regulation between these two gene domains. A further examination of *MADS* gene expression during the transition from vegetative to reproductive growth stages in bamboo revealed distinct patterns of expression (**Figure 4B**). The baseline for this experiment was set using 3-week-old seedlings. The results demonstrated that genes such as *PeMADS63*, *PeMADS59*, *PeMADS44*, *PeMADS81*, *PeMADS78*, *PeMADS75*, *PeMADS72*, *PeMADS82*, and *PeMADS86* displayed an increased expression across various growth stages, with the expression levels rising as the plant transitioned toward reproductive growth. In contrast, the expression levels of genes *PeMADS61*, *PeMADS58*, and *PeMADS60* decreased during this transition. Notably, genes belonging to the K-box domain exhibited a pronounced expression specifically in floral tissues, further emphasizing the distinct roles of the K-box domain in reproductive development. These findings suggest that the *MADS* gene family plays a critical role in regulating both vegetative and reproductive growth phases in bamboo, with differential expression patterns potentially linked to the domain-specific functions of the genes. However, it should be noted that the functional significance of these expression patterns remains to be validated through a further experimental investigation, including functional genomics approaches. In summary, the results highlight the importance of *MADS* genes in bamboo development and provide insights into how their expression varies across tissues and growth stages, contributing to the regulation of both vegetative and reproductive processes.

### 3.5 Identification and characterization of *MADS* genes involved in flower formation and development in bamboo

**Figure 5:**
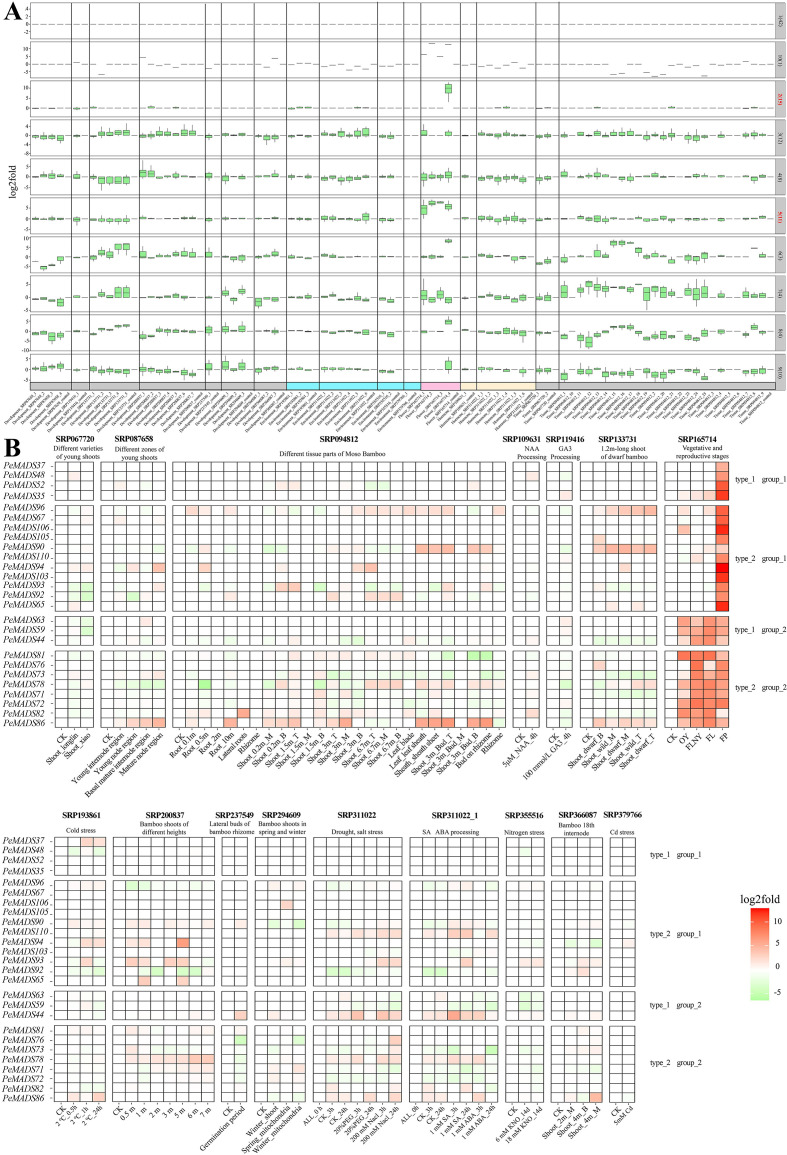
**(A)** *MADS* gene transcriptome expression data of *Phyllostachys edulis* flower. The abscissa is the sample group of different treatments, the ordinate is the gene expression level, and the number of genes in the group parentheses on the right is the number of genes in the group. The color blocks in the abscissa represent different types of samples. The gray group (nine genes) is related to tissue development, the blue group (four genes) is related to environmental stress, the yellow group (two genes) is related to hormone regulation, and the pink group (one gene) is related to flower formation. The key groups discussed in the “Results” are marked in red. **(B)** Transcriptome analysis of *MADS* genes specifically expressed in flowers, with SRA numbers of different transcriptomes at the top, IDs of different genes on the ordinate, and different sample groups on the abscissa. The type_1 on the right represents genes containing only the MADS domain, the type_2 represents genes containing both MADS and K-box domains, group_1 represents the second group of genes on the right side of **(A)**, and group_2 represents the fifth group of genes on the right side of **(A)**. Red represents higher gene expression levels, while green represents lower gene expression levels.

To identify key genes involved in flower formation and developmental regulation, expression data from eight previously analyzed transcriptomic genes were examined (**Figure 5A**). Significant genes were screened and subsequently grouped according to their expression patterns using K-means clustering analysis, which incorporated log2 fold change values. This analysis extended the scope of the transcriptomic investigation and revealed distinct clusters of genes with specific roles in tissue development and hormone regulation. Specifically, the gray group (nine genes) was associated with tissue development, the blue group (four genes) with environmental stress, the yellow group (two genes) with hormone regulation, and the pink group (one gene) with flower formation. Among these, particular attention was given to genes that were exclusively expressed in floral tissues, particularly those from group 2 (15 genes) and group 5 (11 genes), which exhibited a strong expression in tissues related to flower development, including the leaves and flower-related tissues. Additionally, other *MADS* genes were observed to display diverse expression patterns across different tissues—for example, group 6 (three genes), group 7 (four genes), and group 8 (four genes) showed a significant variation in expression among different tissues. Group 1, comprising 42 genes, was not distinguished in the K-means clustering analysis due to data limitations. It should be noted that the clustering analysis results are based on the expression patterns observed in the available transcriptomic data, and further experimental validation is needed to confirm the specific roles of these genes in flower formation and development regulation. From these findings, group 2 and group 5 emerged as particularly significant, with genes from these groups being strongly implicated in flower formation and development regulation. To further investigate, a heatmap analysis was conducted for the 26 genes identified in group 2 and group 5 (**Figure 5B**), which confirmed their high expression levels in the SRP165714 dataset, particularly in flowers at different stages. Notably, the 15 genes from group 2 exhibited the strongest expression in flower florets, with no expression detected in other tissues. In contrast, the 11 genes from group 5 were expressed in 1-year-old leaves (OY) and other flower-related tissues, although their specificity was not as pronounced as that observed in group 2. Based on these observations, it can be hypothesized that the 15 genes in group 2 play a central role in regulating flower formation and development, but further functional studies are required to fully elucidate their roles in this process.

Combining the results of previous stress and hormone expression pattern analyses, it was found that some of the genes playing key roles in flower formation and development simultaneously possess significant stress response and hormone responsiveness. Among them, *PeMADS94*, *PeMADS37*, and *PeMADS93* were induced to upregulate their expression under abiotic stress, while *PeMADS90* and *PeMADS110* showed upregulated expression under salicylic acid (SA) treatment. These findings fully indicate that some members of the *MADS-box* gene family are not solely involved in stress response or hormone transduction but are also associated with the flowering regulation process of *P. edulis*.

### 3.6 Identification of *MADS-box* regulated genes in bamboo flower formation

To gain a deeper understanding of the molecular mechanisms regulating flowering in bamboo, this study was designed to perform a specialized transcriptome analysis across several key developmental stages. The analysis utilized five distinct plant samples: TW (3-week-old seedlings), OY (1-year-old plants), FLNY (plants that are set to flower in the next year), FL (flowering plants), and FP (flower florets). The initial hypothesis was based on the assumption that, with TW as the control, no significant changes in gene expression would be observed between the TW and OY samples, as these plants are not yet undergoing flowering and thus are not expected to exhibit substantial developmental changes. In contrast, significant gene expression changes related to flowering were expected in the FLNY sample, which marks the transition toward flowering in bamboo. However, it should be noted that the assumption of minimal gene expression changes between TW and OY is based on the current understanding of bamboo development, and further experimental validation is needed to confirm this hypothesis. Given that bamboo is a continuously flowering species, it was further hypothesized that the regulatory genes controlling flowering would remain consistent between the FLNY and FL samples, as these plants continuously produce flowers. This hypothesis is built on the premise that flowering regulation in bamboo remains stable during ongoing flowering, though variations across stages could still be present and warrant further investigation. The primary goal of this study was to identify genes regulated by *MADS-box* transcription factors, which are known to be central to flower development.

**Figure 6:**
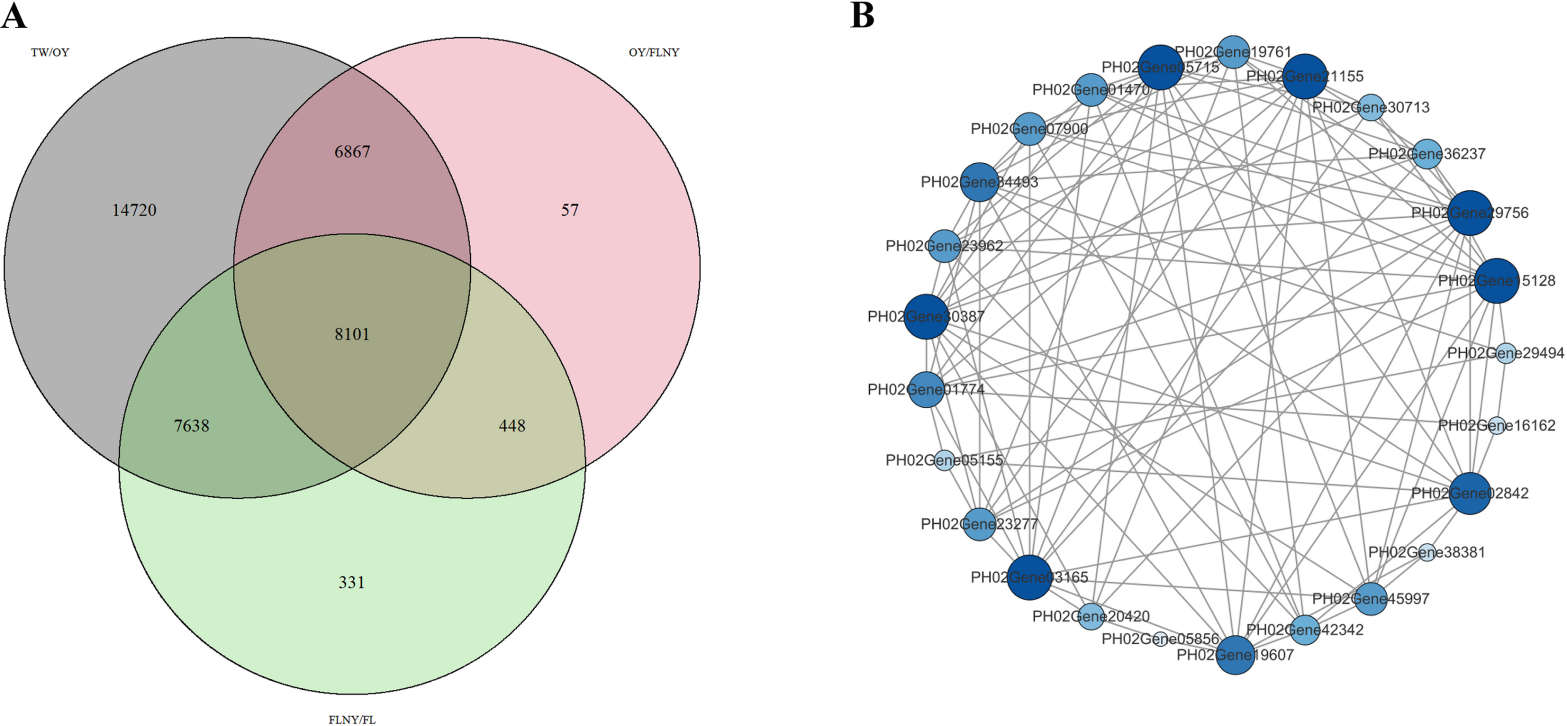
**(A)** TW, 3-week seedling; OY, annual plant; FLNY, second-year-flowering plant; FL, flowering plant; FP, floret. **(B)** Association analysis of related genes expressed in flowers. Several special proteins: PH02Gene21155 belongs to the TRAFAC class myosin-kinesin ATPase superfamily. Kinesin family: PH02Gene30387 belongs to the protein kinase superfamily. Tyr protein kinase family: PH02Gene29756 belongs to the protein kinase superfamily. PH02Gene03165—transcription factor GAMYB. PH02Gene34493 belongs to the TRAFAC class myosin-kinesin ATPase superfamily. Kinesin family: PH02Gene15128 belongs to the TRAFAC class myosin-kinesin ATPase superfamily. Kinesin family: PH02Gene05715—SNAP25 homologous protein.

To test these hypotheses, differential gene expression was analyzed across the different samples, and the data are visualized in **Figure 6A**. This figure highlights genes that showed no significant change between TW and OY (marked in gray), while genes with more than a twofold expression difference between OY/FLNY (pink) and FLNY/FL (green) were identified as significantly regulated during the transition to flowering. It is important to note that while these expression changes were statistically significant, further validation is necessary to confirm their functional relevance in flowering regulation. A Venn diagram was employed to focus on the genes of interest, ultimately selecting 8,101 genes for further analysis. Among these, 15 bamboo MADS genes specifically expressed in flower tissues were identified, and their interactions with other genes were explored, yielding the top 100 gene interactions (**Figure 6B**). These interactions include genes from the TRAFAC class myosin-kinesin ATPase superfamily, particularly the kinesin family, involved in intracellular transport and molecular movement, and tyrosine protein kinases, which are crucial for regulating cellular processes such as signal transduction and cell division. The analysis also identified GAMYB, a transcription factor involved in gene regulation, particularly in plants, further suggesting a connection between these genes and *MADS-box* transcription factors. However, the exact role of GAMYB in the context of bamboo flowering remains to be fully elucidated, and its direct interaction with MADS-box genes should be investigated in future studies. Additionally, the presence of a SNAP25 homologous protein was observed, indicating its role in synaptic transmission and vesicle fusion, which may contribute to the regulatory processes during flowering. Moreover, a *MYB* transcription factor was identified, which is thought to regulate *MADS* genes and play a role in coordinating the developmental processes involved in flowering. These findings provide a preliminary basis for further experimental investigation into the regulatory networks governing flowering in bamboo. Based on these findings, it is hypothesized that these genes are regulated by *MADS-box* transcription factors and interact within the broader gene networks that govern flowering and floral development in bamboo.

### 3.7 Identification of unique *MADS* genes in *P. edulis* through a comparative analysis with other bamboo species

**Figure 7:**
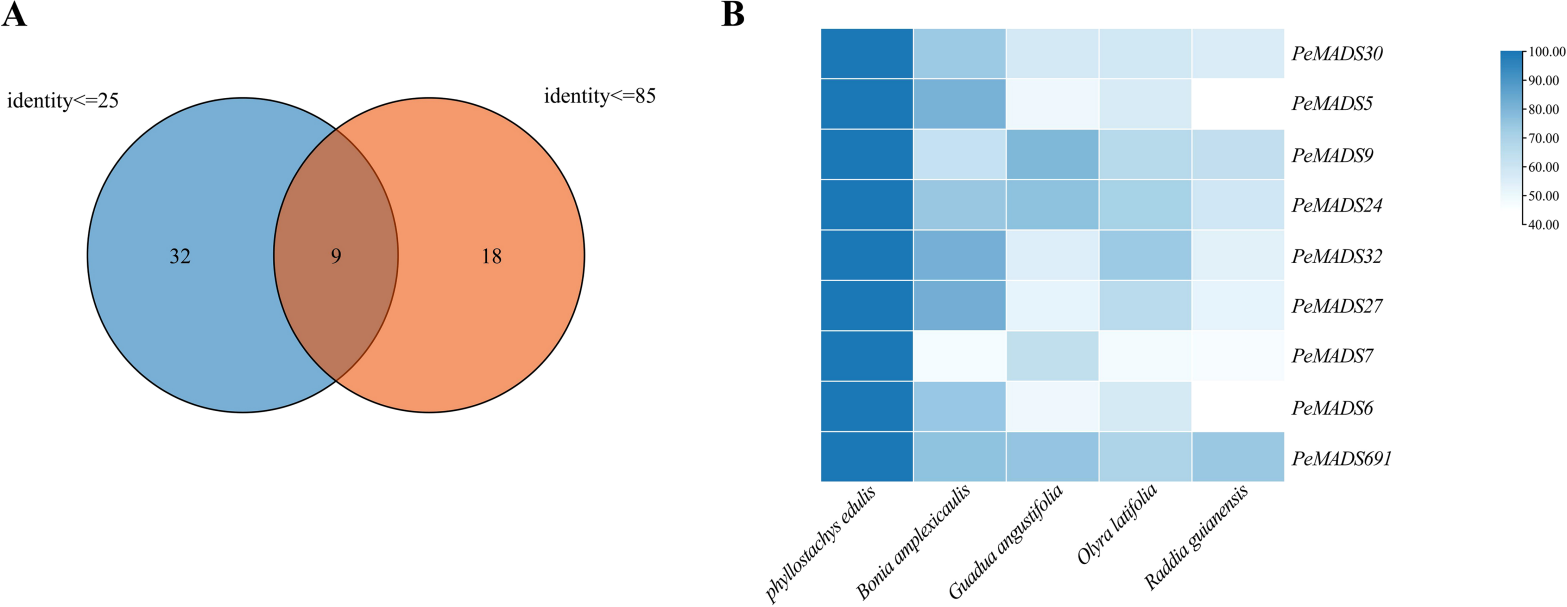
**(A)** The blue part is the gene of moso *Phyllostachys* and four other bamboo MADS genes Identify<=25, and the orange part is the genes of moso *Phyllostachys* and the other four bamboo MADS genes Identify<=85. The intersection of the two is the MADS gene specific to moso bamboo. **(B)** The darker the colorshade of blue, the higher the similarity, and the first column is the gene of moso bamboo, so the colorshade is the darkest.

Understanding the genetic basis of species-specific traits in bamboo species is essential for advancing both basic research and practical applications, such as improving bamboo cultivation and breeding programs. In this study, a comparative analysis of *MADS* genes from *P. edulis* (moso bamboo) and four other bamboo species—*Bonnia amplexicaulis*, *Guadua angustifolia*, *Olyra latifolia*, and *Raddia guianensis*—was conducted to identify the unique *MADS* genes of *P. edulis*. The results of this comparison were systematically processed using an Excel spreadsheet. Initially, genes with an identity score ≤25% were selected as candidates for further analysis. Genes exhibiting an identity score greater than 85% were subsequently excluded from the dataset, as their high similarity to other species’ *MADS* genes would not contribute to the identification of species-specific genes. The remaining genes, those with an identity score ≤85%, were retained for further scrutiny. It should be noted that this approach assumes that similarity scores are an appropriate indicator of species specificity, which should be validated by additional functional analysis. To refine the gene set, a Venn diagram was constructed to intersect the sets of genes with identity scores ≤25% and ≤85%, with the intersection representing the unique *MADS* genes of *P. edulis* (**Figure 7A**). This approach ensured that genes with similarity exceeding 85% were not included in the group defined by ≤25% similarity, thereby focusing on a distinctive set of *MADS* genes specific to *P. edulis*. The resulting unique *MADS* genes of *P. edulis* included *PeMADS5*, *PeMADS6*, *PeMADS7*, *PeMADS9*, *PeMADS24*, *PeMADS27*, *PeMADS30*, *PeMADS32*, and *PeMADS91*, totaling nine genes. To further validate the accuracy of the selection process, the identity scores of these genes were visualized using a heatmap. The heatmap was constructed based on the maximum identity values between the unique *MADS* genes of *P. edulis* and those from the other four bamboo species (**Figure 7B**). The heatmap analysis revealed that the identity scores for the *MADS* genes from the other bamboo species were all below 85%, indicating a relatively low level of similarity between the species. This visual representation confirmed that the selected genes from *P. edulis* are distinct, with minimal overlap in identity with the *MADS* genes from the other bamboo species. Overall, this study provides a robust method for identifying species-specific *MADS* genes, offering valuable insights for future genetic studies and potential biotechnological applications.

### 3.8 Tissue-specific expression of *MADS* genes in *P. edulis*

**Figure 8:**
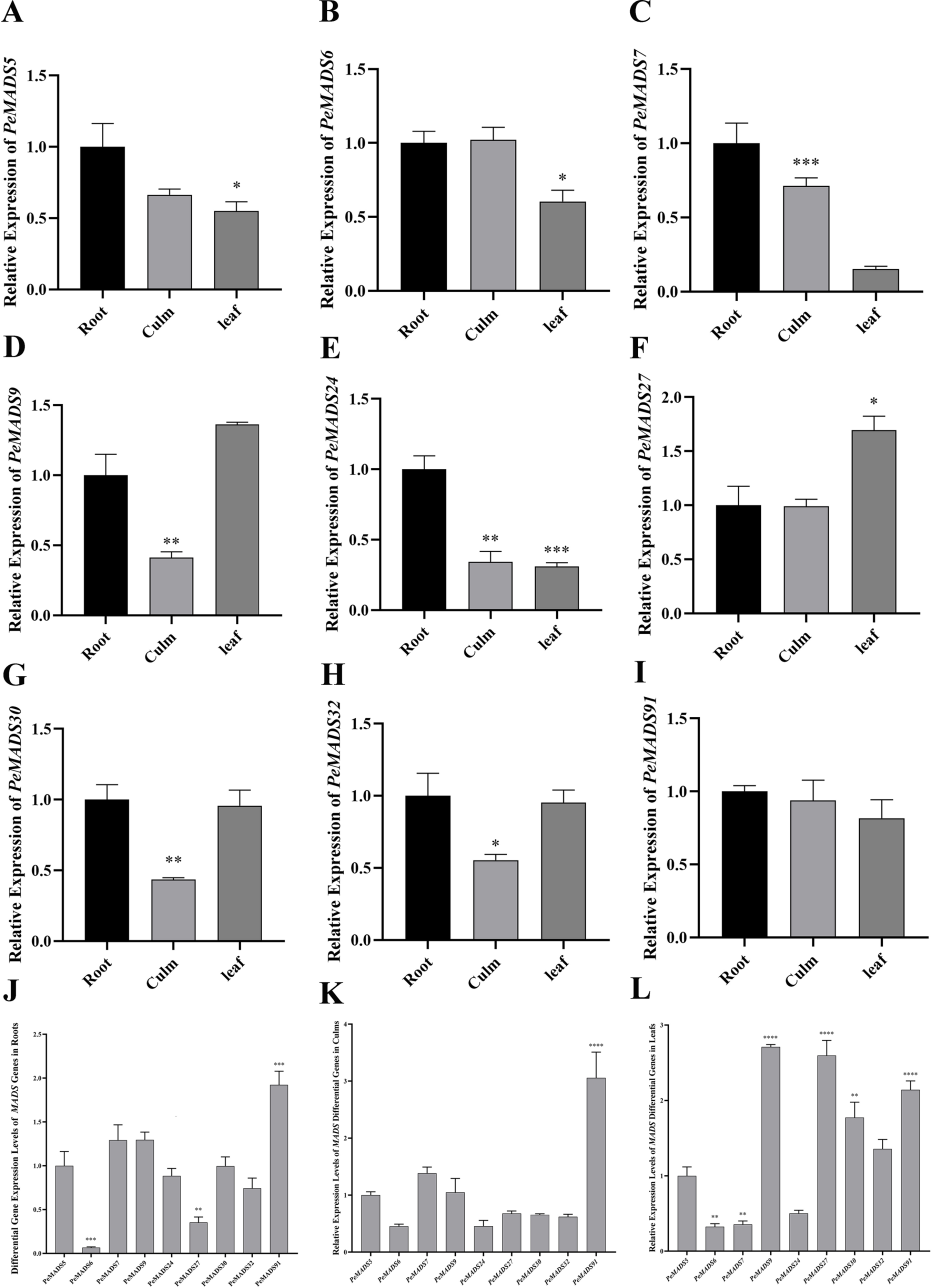
**(A–I)** Relative expression levels of *PeMADS5*, *PeMADS6*, *PeMADS7*, *PeMADS9*, *PeMADS24*, *PeMADS27*, *PeMADS30*, *PeMADS32*, and *PeMADS91* in different tissues of *Phyllostachys edulis*, respectively. **(J–L)** Relative expression levels of the nine differential genes (*PeMADS5*, *PeMADS6*, *PeMADS7*, *PeMADS9*, *PeMADS24*, *PeMADS27*, *PeMADS30*, *PeMADS32*, and *PeMADS91*) in the roots, culms, and leaves of *P. edulis*, respectively. *P<0.05, **P<0.01, ***P<0.001, ****P<0.0001.

To investigate the expression patterns of nine *MADS*-specific genes in different organs of *P. edulis*, real-time quantitative PCR (qPCR) was conducted to analyze the expression levels of these genes in the roots, stems, and leaves of 3-month-old *P. edulis* seedlings. *NTB* was used as the internal reference gene, and three biological replicates were performed. With the expression level in roots as the reference, the results revealed that *PeMADS7*, *PeMADS9*, *PeMADS24*, *PeMADS30*, and *PeMADS32* exhibited a relatively low expression in stems, while *PeMADS9* and *PeMADS27* showed a relatively high expression in leaves (*P* < 0.05). Notably, *PeMADS91* had higher expression levels in roots and stems compared with other *MADS*-specific genes, suggesting that this gene may play a regulatory role in the physiological processes of roots and stems, such as nutrient uptake and translocation. In addition, *PeMADS9* displayed the highest expression level in leaves among all tested genes, indicating its potential regulatory function in leaf physiological activities (**Figure 8**). These data provide a useful basis for subsequent studies on genes in *P. edulis*.

## 4 Discussion

Bamboo, particularly moso bamboo (*P. edulis*), is a remarkable plant species known for its rapid growth and unique reproductive behavior. Unlike most other plants, bamboo experiences long vegetative periods before flowering once in its lifetime, a phenomenon known as gregarious flowering. This unique growth cycle has sparked significant interest in understanding the molecular mechanisms that regulate its development and flowering patterns. *MADS-box* genes, which are known to play critical roles in floral organ development and plant growth, are hypothesized to be key players in regulating these processes. In light of this, our study aims to expand on the findings of Zhang et al. (2018b) by offering a more comprehensive analysis of the *MADS-box* gene family in moso bamboo, delving deeper into its phylogenetic relationships, gene duplication events, and the role of these genes in bamboo’s exceptional growth and reproductive cycles. In comparison to the work by Zhang et al. (2018b), which identified 42 *MADS-box* genes, our study discovered a significantly larger set of 110 *MADS-box* genes in moso bamboo. This expansion of gene families provides a more thorough catalog of the *MADS-box* gene family and allows for a deeper understanding of the functional diversification of these genes. Through phylogenetic analysis, we observed a distinct evolutionary pattern within the bamboo *MADS-box* gene family, highlighting gene duplication events that appear to contribute to bamboo’s unique growth characteristics, such as its ability to thrive in diverse environments and its distinctive flowering cycle. While Zhang et al. (2018b) focused primarily on the role of the *PeMADS5* gene in floral development, our study extends this by examining additional key genes and their roles in regulating both vegetative and reproductive growth stages. Our analysis uncovers how these genes contribute to bamboo’s long vegetative periods and rare flowering cycles, offering new insights into the molecular mechanisms that govern bamboo’s development (Zhang et al., 2018b).

An important aspect of our study is the expansion of the *MADS-box* gene family in bamboo, which significantly contributes to the plant’s adaptability and specialized phenotypic traits. Bamboo’s rapid growth and remarkable adaptability are, in part, a result of this gene family expansion, which increases genetic diversity and functional capacity. As a clonal plant, bamboo exhibits a high degree of phenotypic plasticity, allowing it to thrive in a variety of environmental conditions (Vergara-Silva et al., 2000). Studies have shown that the *MADS-box* gene family plays a crucial role in regulating essential traits such as flower development and stress responses, both of which are critical for bamboo’s survival (Thangavel and Nayar, 2018). Our findings suggest that the expansion of the *MADS-box* gene family in bamboo enhances its ability to adapt to environmental changes and regulate its unique flowering behavior. This expansion is particularly associated with the development of bamboo’s specialized root system, enabling it to survive in a diverse range of habitats (Nezhdanova, 2021). We also observed that several of the newly identified genes are associated with stress responses, particularly those related to environmental fluctuations such as drought and temperature changes, which may be crucial for bamboo’s ability to withstand various ecological challenges.

When comparing the evolution of bamboo’s *MADS-box* gene family to that of other grasses, we observe both similarities and significant differences. Gene duplications in bamboo resemble those seen in other grasses, where *MADS-box* genes regulate key aspects of reproductive development, such as flowering time, inflorescence structure, and stress responses (Jian_Wu, 2002). However, bamboo displays a more extensive expansion of its *MADS-box* gene family, which may be attributed to its unique ecological niche and growth habits. While other grasses tend to have more predictable flowering cycles, bamboo’s expanded *MADS-box* family may facilitate the rapid flowering observed under specific environmental conditions, a behavior not commonly seen in other grasses. This molecular evolution of *MADS-box* genes in bamboo is intricately tied to its survival strategies. Bamboo has harnessed these genes to develop specialized phenotypic traits such as rapid growth and unusual flowering patterns, offering new insights into plant adaptation mechanisms. These findings not only shed light on bamboo’s ecological success but also expand our broader understanding of gene duplication and functional diversification across plant species (Nezhdanova, 2021; Thangavel and Nayar, 2018). As such, the role of the *MADS-box* gene family in bamboo’s evolutionary development underscores the molecular foundations of its rapid growth and distinctive flowering patterns, with implications for enhancing plant adaptability and ecological success across various species. Additionally, our analysis found that the bamboo *MADS-box* genes show more pronounced gene duplications compared to other grasses, which may help explain the species’ ability to adapt rapidly to diverse environments and sustain long vegetative periods.

Building upon this, *MADS-box* genes play an essential role in regulating plant development, especially in aspects like floral organ development, inflorescence architecture, and flowering time. In bamboo, the involvement of these genes becomes particularly intriguing due to the species’ long and infrequent flowering cycles, which sets it apart from more typical grasses like rice and maize. Understanding the function of *MADS-box* genes in bamboo, especially in moso bamboo (*P. edulis*), is crucial for advancing genetic manipulation strategies to optimize bamboo’s growth and flowering. Bamboo’s *MADS-box* genes, similar to those in other grasses, regulate the transition from vegetative to reproductive growth; however, their role in bamboo’s prolonged flowering cycles appears more complex. This complexity involves intricate interactions with hormonal pathways and environmental signals (Vergara-Silva et al., 2000). Specifically, bamboo’s slow transition to flowering suggests that these genes may be more involved in stress adaptation and long-term growth rather than rapid floral initiation. Studies have shown that *MADS-box* genes, particularly those in the Type I and Type II subfamilies, significantly influence flower initiation by interacting with other signaling pathways, which are essential for bamboo’s rare flowering intervals (Nezhdanova, 2021). This combination of gene duplication and variation opens up possibilities for genetic manipulation to synchronize flowering with environmental conditions, presenting an opportunity for more controlled bamboo cultivation. Moreover, our analysis indicates that the timing and expression of specific *MADS-box* genes in bamboo are influenced by both environmental and internal hormonal cues, underscoring the complexity of bamboo’s reproductive cycle.

In contrast to rice, bamboo’s longer reproductive cycle suggests a distinct regulatory mechanism that may better equip it to adapt to varying environmental conditions (Thangavel and Nayar, 2018). This divergence in flowering behavior is pivotal for developing breeding programs that can manage bamboo’s flowering time, thereby enhancing its resilience to climate change. Research has identified specific *MADS-box* genes in moso bamboo, such as *PheMADS15* and *PeMADS5*, which play crucial roles in regulating the flowering process. The overexpression of these genes in model plants, such as *Arabidopsis*, has been shown to induce early flowering, demonstrating that manipulating these genes could optimize flowering times in bamboo (Cheng et al., 2017). Expression analyses further reveal that bamboo’s *MADS-box* genes, categorized into the MIKCC, MIKC*, Mα, and Mβ subfamilies, exhibit expression patterns similar to those in *Arabidopsis* and rice, supporting the applicability of the ABCDE model of floral organ development to bamboo (Cheng et al., 2017; Zhang et al., 2018b). However, bamboo’s unique developmental trajectory is reflected in the distinct timing and intensity of gene expression, setting it apart from other species. These insights not only deepen our understanding of bamboo’s flowering regulation but also open promising avenues for manipulating these genes to synchronize flowering cycles, thereby bolstering bamboo’s productivity and resilience to environmental stress. Our findings emphasize the necessity of investigating bamboo’s gene expression patterns further, as this could lead to more accurate predictions of flowering and growth cycles in different ecological conditions.

The functional diversification of *MADS-box* genes in bamboo provides substantial potential for crop improvement. Similar to other species, where these genes are targeted to enhance reproductive traits, bamboo’s *MADS-box* genes could be leveraged to improve growth and reproductive timing, offering ecological and economic benefits. Bamboo’s versatile ecological role and economic significance further highlight the importance of understanding its genetic pathways. By optimizing bamboo cultivation, we can foster more sustainable production practices. Ultimately, research on *MADS-box* genes is essential for regulating bamboo’s complex flowering patterns. Continued studies will guide breeding strategies aimed at optimizing growth and flowering, ultimately enhancing both ecological stability and economic productivity (Zhang et al., 2018b; Zhang et al., 2021b).

Bamboo’s unique flowering patterns, characterized by long and infrequent flowering cycles, have long intrigued ecologists and plant breeders. This rare flowering behavior, coupled with bamboo’s ecological and economic importance, underscores the need to understand the genetic mechanisms regulating its flowering. Recent research has shown that *MADS-box* genes, which are known to control floral organ identity and flowering time in a wide range of plants, play a pivotal role in bamboo’s reproductive processes—for instance, *PeMADS2* and *PeMADS5* have been linked to the promotion of the flowering transition, with overexpression studies in model plants like *Arabidopsis* and rice providing strong evidence of their involvement in initiating flowering (Zhang et al., 2018b; Zhang et al., 2021b). Other genes, such as *PheMADS15* and *PvMADS56*, have similarly been associated with regulating flowering time, further emphasizing the critical role of *MADS-box* genes in bamboo’s reproductive cycle (Cheng et al., 2017; Liu et al., 2016). Beyond their role in timing, *MADS-box* genes are also integral to the development of floral organs, following the conserved ABCDE model of floral organogenesis, which holds true in bamboo (Cheng et al., 2017).

Additionally, bamboo’s response to environmental factors such as temperature, light, and stress has been shown to influence the expression of *MADS-box* genes, highlighting the adaptability of bamboo to varying ecological conditions. This adaptability is crucial for ensuring successful growth and reproduction in a changing climate. Our study also identified several *MADS-box* genes that are upregulated during periods of environmental stress, suggesting their potential roles in stress adaptation. The ability to manipulate these genes may open new avenues for improving bamboo’s resilience to climate change and other environmental challenges. Furthermore, these findings hold great promise for developing more sustainable bamboo cultivation practices that align with modern agricultural needs. Understanding the regulatory network of *MADS-box* genes in bamboo could lead to better control over flowering times, which is crucial for managing bamboo resources for industrial and ecological purposes.

A comparative analysis between *P. edulis* (moso bamboo) and four other bamboo species, namely, *Bonia amplexicaulis*, *Guadua angustifolia*, *Olyra latifolia*, and *Raddia guianensis*, successfully identified nine moso bamboo-specific *MADS-box* genes, including *PeMADS5*, *PeMADS6*, *PeMADS7*, *PeMADS9*, *PeMADS24*, *PeMADS27*, *PeMADS30*, *PeMADS32*, and *PeMADS91*; the relative expression levels of these genes further revealed their potential functional roles, with *PeMADS9*, *PeMADS27*, and *PeMADS91* exhibiting relatively high expression levels in leaves (suggesting their involvement in leaf development, vegetative growth, as well as light signal perception and transduction) and *PeMADS91* showing the highest expression levels in roots and stems (indicating its putative function in underground root system development, aerial stem growth, and adaptation to underground abiotic stresses such as drought); notably, current research on the functional validation of these moso bamboo-specific *MADS-box* genes remains scarce, limiting our in-depth understanding of the molecular mechanisms underlying the unique biological characteristics of *P. edulis*, while the nine identified genes provide valuable candidate loci for deciphering these unique life phenomena and filling the research gap in the functional study of moso bamboo-specific transcription factors, and future studies should focus on their functional characterization using gain-of-function and loss-of-function experiments, combined with transcriptomic, metabolomic, and physiological analyses to clarify their downstream target genes and molecular mechanisms, which will not only expand the repertoire of *MADS-box* genes in bamboo species but also provide important genetic resources for moso bamboo molecular breeding, laying a solid foundation for future in-depth studies and promoting the sustainable development of the bamboo industry.

In conclusion, the *MADS-box* gene family in moso bamboo plays an essential role in regulating its reproductive cycle, including its long vegetative periods and infrequent flowering. Our study builds upon previous research by identifying a larger set of *MADS-box* genes and exploring their evolutionary significance, expression patterns, and functional diversification. These findings offer new insights into the molecular mechanisms underlying bamboo’s growth and flowering behavior, with potential applications in bamboo breeding and cultivation. Continued exploration of these genes will be critical for optimizing bamboo’s ecological and economic potential, ensuring its continued importance as a sustainable resource for various industries.

## 5 Conclusion

This study provides a comprehensive analysis of the *PeMADS* gene family in *P. edulis* (moso bamboo), significantly expanding upon previous research by identifying 110 *PeMADS* genes. The classification into Type 1 and Type 2 genes, along with the identification of 15 Type 2 genes exclusively expressed in flower florets, suggests a specialized role in floral development. Our large-scale integration of RNA-seq datasets has not only identified potential *PeMADS*-regulated genes but also revealed a complex regulatory network that could inform future functional studies. Furthermore, the evolutionary comparison of *PeMADS* across different bamboo species highlights both conserved and species-specific evolutionary patterns, deepening our understanding of the molecular evolution of *MADS-box* genes in bamboo. This study lays a crucial foundation for future research into the functional roles of *PeMADS* genes and their application in bamboo breeding and stress management.

## Data Availability

The genome sequence of *Phyllostachys edulis* (Moso bamboo) was downloaded from the GIGADB DATASETS repository (http://gigadb.org/dataset/view/id/100498/File_page/2). The database is recognized as an authoritative database at home and abroad, and the data quality is widely recognized. The genome sequences of Bonia amplexicaulis, Guadua angustifolia, Olyra latifolia, and Raddia guianensis were obtained from the Bamboo Genome Database (http://www.genobank.org/bamboo#2). Sixteen sets of transcriptome data for Moso bamboo were retrieved from NCBI, with the raw RNA data obtained from the NCBI Sequencing Read Archive (SRA, https://www.ncbi.nlm.nih.gov/sra). The project numbers are SRP311022, SRP087658,193861, SRP379766, SRP355516, SRP133731, SRP294609, SRP119416, SRP200837, SRP366087, SRP237549, SRP109631, SRP311022_1, SRP067720, SRP165714, SRP094812, respectively.
