# Supplementary material for: Comprehensive characterization of the PeMADS gene family in Phyllostachys edulis reveals new insights into floral development and evolution
Source: Front Plant Sci. 2026 Apr 21;17:1806675. doi: 10.3389/fpls.2026.1806675 (PMC13139355; doi:10.3389/fpls.2026.1806675)
Supplement: Supplementary file 1 [file Table1.docx]

Supplementary Table 1:Primers for qRT-PCR of PeMADS differentially expressed genes

| **Gene Name** | **Primer Type** | **Primer Sequence (5'→3')** | **Purpose** |
| --- | --- | --- | --- |
| PeMADS5 | Forward Primer (F) | CTTTGGCAAGGTAGAGGAGC | qRT-PCR detection of target genes |
| PeMADS5 | Reverse Primer (R) | GTCGCAGCGGAAATAGATG | qRT-PCR detection of target genes |
| PeMADS6 | Forward Primer (F) | GAACCCGCTGCTATCACAAG | qRT-PCR detection of target genes |
| PeMADS6 | Reverse Primer (R) | GCCGAAGTTAGGAATGGAGG | qRT-PCR detection of target genes |
| PeMADS7 | Forward Primer (F) | GCATCGAGGACGACCGTAA | qRT-PCR detection of target genes |
| PeMADS7 | Reverse Primer (R) | AGGGCTTGCCAAAGGTGA | qRT-PCR detection of target genes |
| PeMADS9 | Forward Primer (F) | CTCCTCCGCATTTCAACCA | qRT-PCR detection of target genes |
| PeMADS9 | Reverse Primer (R) | CAGCTTAGCGGCCTTCCAT | qRT-PCR detection of target genes |
| PeMADS24 | Forward Primer (F) | ATCAAGCCCATCCAGAACG | qRT-PCR detection of target genes |
| PeMADS24 | Reverse Primer (R) | ACAGCAACTTCTGCACCACATAGGA | qRT-PCR detection of target genes |
| PeMADS27 | Forward Primer (F) | GTTGCCAGACGATGACTACGC | qRT-PCR detection of target genes |
| PeMADS27 | Reverse Primer (R) | TAGCCGTCCATGTCCACCA | qRT-PCR detection of target genes |
| PeMADS30 | Forward Primer (F) | GACAACCGTGAGCGTGAGA | qRT-PCR detection of target genes |
| PeMADS30 | Reverse Primer (R) | AGCAAGAGGTTCTACGTCCAT | qRT-PCR detection of target genes |
| PeMADS32 | Forward Primer (F) | TGATGAAGAAGGCGAAAGAGC | qRT-PCR detection of target genes |
| PeMADS32 | Reverse Primer (R) | AAGCGTTGGAGGATGGTTGT | qRT-PCR detection of target genes |
| PeMADS91 | Forward Primer (F) | GTCGGCGTCGTCATCTTCT | qRT-PCR detection of target genes |
| PeMADS91 | Reverse Primer (R) | AGCTGGGTGTTGTTGGTGG | qRT-PCR detection of target genes |
| PeNTB | Forward Primer (F) | TCTTGTTTGACACCGAAGAGGAG | Internal reference gene (normalization) |
| PeNTB | Reverse Primer (R) | AATAGCTGTCCCTGGAGGAGTTT | Internal reference gene (normalization) |

*PeNTB* was used as the internal reference gene for qRT-PCR normalization, which is a stably expressed housekeeping gene in *Phyllostachys edulis.*
