# Supplementary material for: Comprehensive characterization of the PeMADS gene family in Phyllostachys edulis reveals new insights into floral development and evolution
Source: Front Plant Sci. 2026 Apr 21;17:1806675. doi: 10.3389/fpls.2026.1806675 (PMC13139355; doi:10.3389/fpls.2026.1806675)
Supplement: Supplementary file 2 [file Table2.docx]

Supplementary Table 2: The results of physiochemical analysis of MADS protein in *Phyllostachys edulis*.

| **Name** | **Sequence ID** | **Number of Amino Acid（aa）** | **Molecular Weight（Da）** | **Theoretical pI** | **Instability Index** | **Aliphatic Index** | **Grand Average of Hydropathicity** |
| --- | --- | --- | --- | --- | --- | --- | --- |
| PeMADS1 | PH02Gene45747.t1 | 164 | 17696.93 | 9.65 | 61.56 | 63.78 | -0.6 |
| PeMADS2 | PH02Gene15690.t1 | 197 | 21398.4 | 10.51 | 56.8 | 74.42 | -0.313 |
| PeMADS3 | PH02Gene34343.t1 | 216 | 23750.94 | 6.37 | 45.44 | 87.59 | -0.236 |
| PeMADS4 | PH02Gene29782.t1 | 184 | 20377.26 | 8.42 | 35.55 | 91.68 | -0.298 |
| PeMADS5 | PH02Gene20194.t1 | 452 | 49159.78 | 4.78 | 69.23 | 72.79 | -0.589 |
| PeMADS6 | PH02Gene20193.t1 | 419 | 45675.13 | 5.01 | 67.46 | 77.11 | -0.577 |
| PeMADS7 | PH02Gene45449.t1 | 151 | 16676.78 | 6.75 | 79.7 | 71.13 | -0.487 |
| PeMADS8 | PH02Gene34677.t1 | 381 | 40744.16 | 9.71 | 69.86 | 82.62 | -0.111 |
| PeMADS9 | PH02Gene02129.t1 | 549 | 60008.24 | 9.36 | 50.06 | 69.23 | -0.587 |
| PeMADS10 | PH02Gene36922.t1 | 158 | 17728.73 | 10.78 | 57.05 | 95.13 | -0.382 |
| PeMADS11 | PH02Gene35597.t1 | 154 | 17320.81 | 9.6 | 61.48 | 79.22 | -0.506 |
| PeMADS12 | PH02Gene35593.t1 | 702 | 79168.4 | 8.94 | 53.35 | 78.8 | -0.461 |
| PeMADS13 | PH02Gene01436.t1 | 199 | 22009.97 | 9.26 | 67.98 | 75.18 | -0.553 |
| PeMADS14 | PH02Gene35590.t1 | 157 | 17519.2 | 10.57 | 48.86 | 79.75 | -0.434 |
| PeMADS15 | PH02Gene35596.t1 | 256 | 28992.51 | 9.78 | 55.06 | 86.99 | -0.361 |
| PeMADS16 | PH02Gene35592.t1 | 222 | 25335.06 | 10.09 | 48.84 | 74.37 | -0.681 |
| PeMADS17 | PH02Gene20058.t1 | 361 | 40095.16 | 9.36 | 31.43 | 89.72 | -0.296 |
| PeMADS18 | PH02Gene28445.t1 | 226 | 23995.3 | 8.54 | 61.78 | 81.81 | -0.08 |
| PeMADS19 | PH02Gene28398.t1 | 192 | 20378.32 | 9.55 | 64.65 | 83.07 | -0.074 |
| PeMADS20 | PH02Gene28399.t1 | 264 | 27853.84 | 8.88 | 66.46 | 89.92 | -0.117 |
| PeMADS21 | PH02Gene25812.t1 | 329 | 35992.1 | 8.78 | 57.58 | 69.09 | -0.426 |
| PeMADS22 | PH02Gene25816.t1 | 228 | 25417.83 | 6.37 | 38.17 | 67.11 | -0.567 |
| PeMADS23 | PH02Gene31370.t1 | 252 | 27533.4 | 7.78 | 46.89 | 71.87 | -0.388 |
| PeMADS24 | PH02Gene31369.t1 | 244 | 26811.63 | 6.66 | 48.68 | 74.3 | -0.364 |
| PeMADS25 | PH02Gene13574.t1 | 417 | 44876.7 | 4.24 | 43.45 | 78.87 | -0.533 |
| PeMADS26 | PH02Gene37724.t1 | 752 | 82380.28 | 5.54 | 48.19 | 84.36 | -0.46 |
| PeMADS27 | PH02Gene32821.t1 | 379 | 40876.72 | 4.69 | 28.15 | 70.05 | -0.441 |
| PeMADS28 | PH02Gene04916.t1 | 471 | 50937.63 | 4.77 | 30.9 | 70.04 | -0.508 |
| PeMADS29 | PH02Gene04306.t1 | 385 | 42296.82 | 5.9 | 55.52 | 99.17 | -0.138 |
| PeMADS30 | PH02Gene43712.t1 | 249 | 27922.33 | 9.35 | 55.69 | 90.88 | -0.393 |
| PeMADS31 | PH02Gene27727.t1 | 265 | 28914.09 | 6.2 | 48.43 | 73.66 | -0.386 |
| PeMADS32 | PH02Gene24668.t1 | 275 | 30418.32 | 9.33 | 52.1 | 76.65 | -0.356 |
| PeMADS33 | PH02Gene42439.t1 | 262 | 28751.38 | 9.88 | 57.86 | 76.79 | -0.395 |
| PeMADS34 | PH02Gene08604.t1 | 104 | 12100.11 | 10.04 | 36.8 | 84.42 | -0.282 |
| PeMADS35 | PH02Gene19607.t1 | 142 | 15993.47 | 9.73 | 40.72 | 76.9 | -0.299 |
| PeMADS36 | PH02Gene03359.t1 | 71 | 8098.46 | 9.78 | 42.82 | 86.48 | -0.107 |
| PeMADS37 | PH02Gene29865.t1 | 93 | 10468.01 | 9.45 | 45.54 | 80.75 | -0.332 |
| PeMADS38 | PH02Gene50969.t1 | 61 | 6949.15 | 10.36 | 47.92 | 95.9 | -0.198 |
| **Name** | **Sequence ID** | **Number of**  **Amino Acid（aa）** | **Molecular Weight**  **（Da）** | **Theoretical pI** | **Instability Index** | **Aliphatic Index** | **Grand Average of Hydropathicity** |
| PeMADS39 | PH02Gene41977.t1 | 91 | 10196.8 | 9.94 | 36.91 | 85.71 | -0.37 |
| PeMADS40 | PH02Gene08115.t1 | 92 | 10342 | 9.94 | 43.55 | 91.09 | -0.34 |
| PeMADS41 | PH02Gene15248.t1 | 75 | 8246.59 | 10.69 | 49.17 | 88.4 | -0.271 |
| PeMADS42 | PH02Gene15247.t1 | 75 | 8246.59 | 10.69 | 49.17 | 88.4 | -0.271 |
| PeMADS43 | PH02Gene38054.t1 | 69 | 7906.31 | 10.37 | 41.61 | 77.68 | -0.307 |
| PeMADS44 | PH02Gene22583.t1 | 63 | 7121.4 | 10.65 | 45.77 | 86.67 | -0.148 |
| PeMADS45 | PH02Gene45402.t1 | 93 | 10426.05 | 9.74 | 51.52 | 82.8 | -0.418 |
| PeMADS46 | PH02Gene10417.t2 | 66 | 7501.91 | 10.01 | 47.84 | 121.21 | 0.374 |
| PeMADS47 | PH02Gene46663.t1 | 360 | 40261.38 | 6.27 | 56.6 | 71.64 | -0.631 |
| PeMADS48 | PH02Gene29494.t1 | 290 | 32105.5 | 7.12 | 62.98 | 68.72 | -0.503 |
| PeMADS49 | PH02Gene06936.t1 | 108 | 12168.23 | 10.56 | 63.75 | 91.3 | -0.323 |
| PeMADS50 | PH02Gene23870.t1 | 325 | 35559.96 | 4.71 | 50.97 | 77.2 | -0.319 |
| PeMADS51 | PH02Gene31235.t1 | 538 | 59244.02 | 9.03 | 51.76 | 87.36 | -0.1 |
| PeMADS52 | PH02Gene23962.t1 | 343 | 38064.3 | 5.04 | 63.42 | 80.47 | -0.328 |
| PeMADS53 | PH02Gene08582.t1 | 348 | 39283.52 | 5.12 | 69.64 | 78.16 | -0.418 |
| PeMADS54 | PH02Gene07255.t1 | 165 | 18294.44 | 9.73 | 62.82 | 68.73 | -0.813 |
| PeMADS55 | PH02Gene05780.t1 | 165 | 18363.29 | 9.41 | 59.37 | 63.39 | -0.927 |
| PeMADS56 | PH02Gene37412.t1 | 127 | 14530.3 | 10 | 84.21 | 65.28 | -0.816 |
| PeMADS57 | PH02Gene37413.t1 | 215 | 24276.41 | 9.47 | 71.36 | 78.56 | -0.63 |
| PeMADS58 | PH02Gene08606.t1 | 204 | 23077.95 | 6.65 | 68.5 | 73.19 | -0.691 |
| PeMADS59 | PH02Gene37416.t1 | 183 | 20835.7 | 6.96 | 64.72 | 78.85 | -0.537 |
| PeMADS60 | PH02Gene43392.t1 | 209 | 23800.98 | 8.73 | 67.01 | 73.25 | -0.699 |
| PeMADS61 | PH02Gene08608.t1 | 294 | 32266.26 | 5.12 | 65.18 | 94.12 | -0.43 |
| PeMADS62 | PH02Gene24701.t1 | 106 | 12344.32 | 10.03 | 66.84 | 78.11 | -0.623 |
| PeMADS63 | PH02Gene50874.t1 | 130 | 14855.95 | 10.06 | 62.77 | 72.69 | -0.695 |
| PeMADS64 | PH02Gene26979.t1 | 96 | 11036.81 | 9.69 | 43.72 | 80.21 | -0.662 |
| PeMADS65 | PH02Gene01470.t1 | 248 | 28305.32 | 8.9 | 42.68 | 81.85 | -0.649 |
| PeMADS66 | PH02Gene21703.t1 | 347 | 39429.37 | 9.11 | 53.47 | 84.38 | -0.547 |
| PeMADS67 | PH02Gene17230.t1 | 176 | 20590.79 | 9.49 | 66.45 | 88.64 | -0.622 |
| PeMADS68 | PH02Gene37411.t1 | 246 | 28596.63 | 8.61 | 54.22 | 78.5 | -0.764 |
| PeMADS69 | PH02Gene36237.t1 | 246 | 28170.03 | 9.01 | 51.46 | 79.31 | -0.718 |
| PeMADS70 | PH02Gene11089.t1 | 240 | 27096.84 | 6.26 | 68.51 | 93.08 | -0.482 |
| PeMADS71 | PH02Gene20385.t3 | 263 | 29352.07 | 9.01 | 53.83 | 81.22 | -0.732 |
| PeMADS72 | PH02Gene16543.t1 | 257 | 28989.63 | 9.43 | 53.15 | 75.53 | -0.878 |
| PeMADS73 | PH02Gene30811.t3 | 214 | 24931.81 | 9.04 | 52.88 | 93.32 | -0.648 |
| PeMADS74 | PH02Gene21706.t1 | 245 | 28234.41 | 9.2 | 54.42 | 81.59 | -0.738 |
| PeMADS75 | PH02Gene18087.t1 | 259 | 29839.12 | 9.23 | 63.27 | 78.38 | -0.795 |
| PeMADS76 | PH02Gene42323.t2 | 254 | 29281.54 | 9.24 | 67.22 | 75.71 | -0.807 |
| PeMADS77 | PH02Gene38348.t1 | 254 | 28444.38 | 8.92 | 54.22 | 79.21 | -0.718 |
| PeMADS78 | PH02Gene29534.t1 | 245 | 27778.83 | 9.57 | 53.71 | 88.41 | -0.701 |
| **Name** | **Sequence ID** | **Number of**  **Amino Acid** | **Molecular Weight** | **Theoretical pI** | **Instability Index** | **Aliphatic Index** | **Grand Average of Hydropathicity** |
| PeMADS79 | PH02Gene23951.t1 | 212 | 24101.69 | 8.65 | 40.61 | 89.2 | -0.628 |
| PeMADS80 | PH02Gene05469.t1 | 230 | 26035.64 | 7.76 | 56.2 | 88.26 | -0.57 |
| PeMADS81 | PH02Gene49644.t2 | 226 | 25263.44 | 5.26 | 57.21 | 82.88 | -0.58 |
| PeMADS82 | PH02Gene08550.t1 | 224 | 25116.31 | 5.53 | 61.64 | 81.88 | -0.611 |
| PeMADS83 | PH02Gene02550.t1 | 228 | 25690.79 | 5.65 | 59.33 | 85.57 | -0.653 |
| PeMADS84 | PH02Gene10764.t1 | 228 | 25552.64 | 5.4 | 53.82 | 84.25 | -0.646 |
| PeMADS85 | PH02Gene41439.t1 | 229 | 26411.9 | 7.76 | 58.76 | 85.2 | -0.692 |
| PeMADS86 | PH02Gene05306.t1 | 238 | 27354.07 | 8.32 | 58.47 | 82.35 | -0.69 |
| PeMADS87 | PH02Gene15406.t1 | 239 | 27523.21 | 8.89 | 52.37 | 88.12 | -0.72 |
| PeMADS88 | PH02Gene03960.t1 | 240 | 27554.2 | 7.78 | 44.87 | 88.54 | -0.695 |
| PeMADS89 | PH02Gene27480.t1 | 240 | 27475.1 | 7.8 | 41.34 | 85.67 | -0.735 |
| PeMADS90 | PH02Gene21974.t1 | 240 | 27387.94 | 8.82 | 39.2 | 83.25 | -0.765 |
| PeMADS91 | PH02Gene34319.t1 | 247 | 28321.1 | 5.53 | 58.97 | 90 | -0.521 |
| PeMADS92 | PH02Gene01774.t1 | 238 | 27879.09 | 9.03 | 59.08 | 88.11 | -0.563 |
| PeMADS93 | PH02Gene02842.t1 | 222 | 25371.77 | 8.95 | 38.25 | 73.42 | -0.653 |
| PeMADS94 | PH02Gene19761.t1 | 221 | 25371.79 | 8.87 | 38.91 | 71.54 | -0.681 |
| PeMADS95 | PH02Gene07879.t1 | 209 | 24088.49 | 8.76 | 44.59 | 86.32 | -0.764 |
| PeMADS96 | PH02Gene45997.t3 | 242 | 28538.92 | 9.05 | 62.72 | 83.8 | -0.678 |
| PeMADS97 | PH02Gene15521.t1 | 209 | 24534 | 8.48 | 63.36 | 76.99 | -0.878 |
| PeMADS98 | PH02Gene18783.t1 | 196 | 22293.44 | 6.02 | 46.03 | 88.57 | -0.507 |
| PeMADS99 | PH02Gene15341.t1 | 196 | 22504.65 | 7.71 | 49.47 | 88.06 | -0.598 |
| PeMADS100 | PH02Gene05174.t1 | 202 | 23458.14 | 6.45 | 65.74 | 89.8 | -0.482 |
| PeMADS101 | PH02Gene50204.t1 | 223 | 25284.37 | 8.82 | 42.26 | 90.13 | -0.345 |
| PeMADS102 | PH02Gene43573.t1 | 224 | 25411.33 | 6.76 | 44.31 | 91.03 | -0.347 |
| PeMADS103 | PH02Gene07900.t1 | 252 | 28424.21 | 9.24 | 53.62 | 77.18 | -0.602 |
| PeMADS104 | PH02Gene40583.t1 | 250 | 28079.67 | 9.04 | 57 | 77.44 | -0.616 |
| PeMADS105 | PH02Gene23277.t1 | 253 | 28157.15 | 9.21 | 51.76 | 87.35 | -0.444 |
| PeMADS106 | PH02Gene30713.t1 | 253 | 28155.15 | 8.97 | 49.06 | 90.04 | -0.396 |
| PeMADS107 | PH02Gene46575.t1 | 396 | 44915.94 | 8 | 42.8 | 79.34 | -0.512 |
| PeMADS108 | PH02Gene08228.t2 | 263 | 30132.9 | 9.06 | 47.71 | 71.25 | -0.851 |
| PeMADS109 | PH02Gene39489.t3 | 262 | 29768.74 | 8.77 | 62.84 | 78.63 | -0.662 |
| PeMADS110 | PH02Gene20420.t1 | 261 | 29625.52 | 8.97 | 55.72 | 73.68 | -0.734 |
