# Supplementary material for: Comprehensive characterization of the PeMADS gene family in Phyllostachys edulis reveals new insights into floral development and evolution
Source: Front Plant Sci. 2026 Apr 21;17:1806675. doi: 10.3389/fpls.2026.1806675 (PMC13139355; doi:10.3389/fpls.2026.1806675)
Supplement: Supplementary file 3 [file Table3.docx]

| Supplementary Table 3:Conserved motifs identified in moso bamboo MADS-box proteins | | | | | |
| --- | --- | --- | --- | --- | --- |
|  | **Motif ID** | **Amino Acid Sequences** | **Motif Length(aa)** | **Number of Sites** | **E-value** |
| type_1 | Motif1 | JKRIENKTSRQVTFSKRRNGLFKKAYELSVLCDAEVALVVFSPAGKLYEF | 50 | 58 | 9.1e-1725 |
|  | Motif2 | SSNSSIEKIFGRYWDIPNTTNHLNIEARDSRVDCKIQVKHN | 41 | 5 | 1.6e-112 |
|  | Motif3 | GSPSVDAVJDRYLPLSGDDDP | 21 | 29 | 2.2e-96 |
|  | Motif4 | EADVEALGEDELEEFHKALAALQDAVRGRAD | 31 | 13 | 5.7e-84 |
|  | Motif5 | RAAEELRELMAAEKARMERVGEAVEREKA | 29 | 13 | 3.2e-84 |
|  | Motif6 | KQTKKPLPQSHMHTTWASPFLFGGKSVTPMSTTLPSSIYGLHEELDVNSM | 50 | 4 | 4.3e-81 |
|  | Motif7 | MGRQKI | 6 | 47 | 8.6e-68 |
|  | Motif8 | NLBEMSLEEJEKLEKLVDDALRAVKERKK | 29 | 18 | 8.8e-61 |
|  | Motif9 | RFKDLPELDQDKKMLDQEGFLQQKIDKLNEKLHNARRDNRERETNLMIHD | 50 | 4 | 7.6e-42 |
|  | Motif10 | NIEEJQQEIRRYQQQLQJSEERLRLFEPD | 29 | 4 | 5.7e-28 |
| type_2 | Motif1 | MGRGKIEJKRIENKTNRQVTFSKRRNGLLKKAYELSVLCDAEVALIVFSS | 50 | 47 | 5.1e-1770 |
|  | Motif2 | RQLMGEDLESLSVKELQQLEQQLEKSLKKIRSRK | 34 | 42 | 1.8e-499 |
|  | Motif3 | RGKLYEYASNSMKSK | 15 | 46 | 1.1e-226 |
|  | Motif4 | DQLLLEZIEELQRKESLLQEENMELRKKL | 29 | 45 | 4.8e-250 |
|  | Motif5 | QYWQQEAAKLRQQIZNLQNSN | 21 | 34 | 5.2e-132 |
|  | Motif6 | IERYKKASKELSEAD | 15 | 41 | 1.6e-81 |
|  | Motif7 | LTEKVYTEEGQSSESVMSATHYGSSQDNDDGSDVSLKLGLP | 41 | 9 | 4.4e-71 |
|  | Motif8 | ELQPLPPYDNRGYFPPVLM | 19 | 13 | 7.3e-31 |
|  | Motif9 | NEKMLEDENKLLAFRPHPQDVELSGSMRELELGYHQGRDF | 40 | 6 | 2.2e-31 |
|  | Motif10 | PSLDLNLEHSKYASLNEQLAE | 21 | 5 | 3.3e-16 |
