# Supplementary figures and images for: Comprehensive characterization of the PeMADS gene family in Phyllostachys edulis reveals new insights into floral development and evolution
Source: Front Plant Sci. 2026 Apr 21;17:1806675. doi: 10.3389/fpls.2026.1806675 (PMC13139355; doi:10.3389/fpls.2026.1806675)

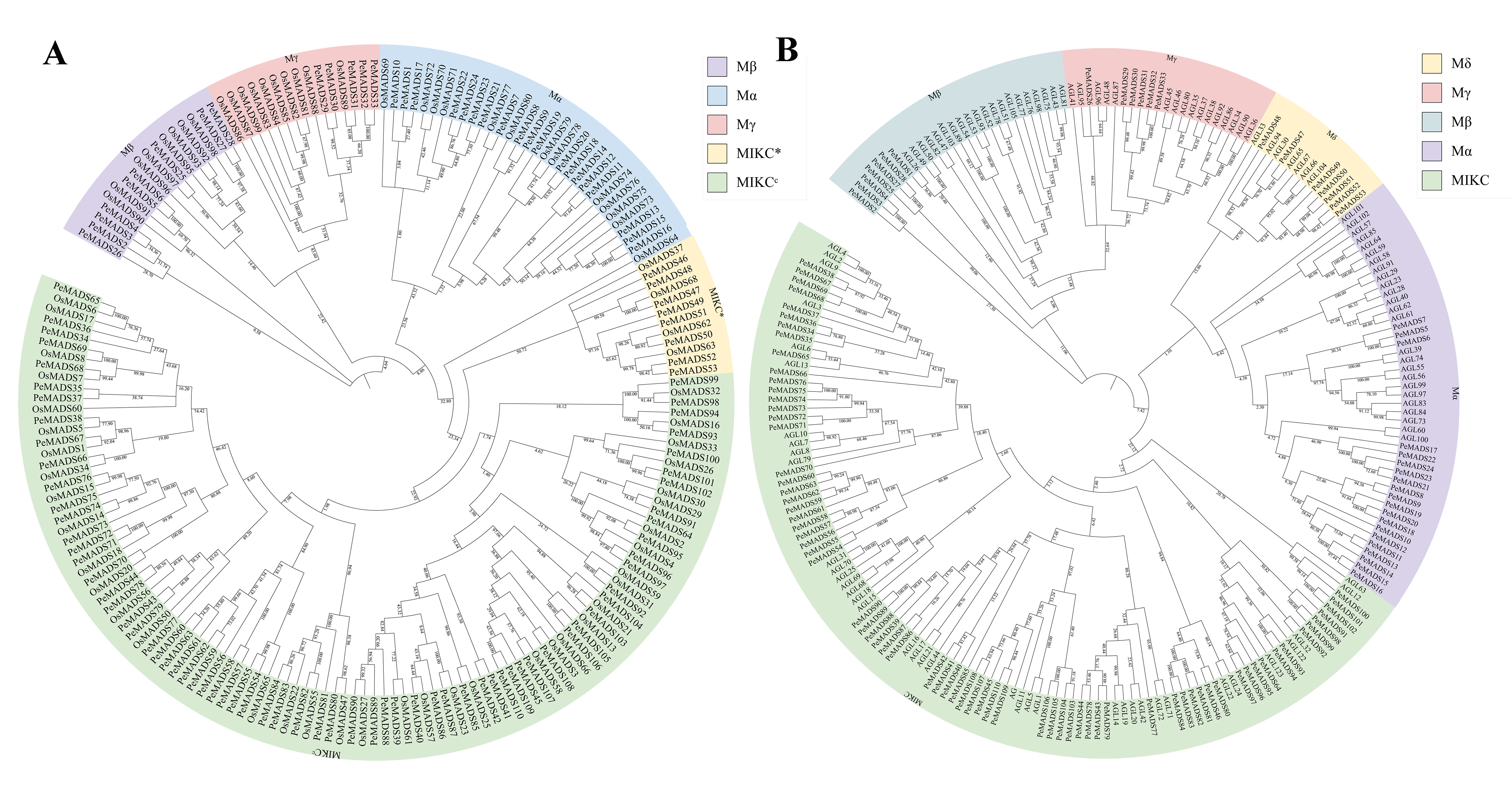

Supplement: Supplementary file 4 [file Image1.jpeg]
